# Flow cytometry-based peripheral blood analysis as an easily friendly tool for prognostic monitoring of acute ischemic stroke: a multicenter study

**DOI:** 10.3389/fimmu.2024.1402724

**Published:** 2024-05-21

**Authors:** Kang Lu, Wanmao Ni, Juanqing Yue, Yongran Cheng, Jing Du, Yanchun Li, Xiangmin Tong, Guo-Bo Chen, Ying Wang

**Affiliations:** ^1^ Department of Medical Laboratory Medicine, The First Affiliated Hospital of Zhejiang Chinese Medical University (Zhejiang Provincial Hospital of Chinese Medicine), Hangzhou, Zhejiang, China; ^2^ Clinical Research Institute, Zhejiang Provincial People’s Hospital, People’s Hospital of Hangzhou Medical College, Hangzhou, Zhejiang, China; ^3^ Department of Pathology, Affiliated Hangzhou First People’s Hospital, School of Medicine, Westlake University, Hangzhou, Zhejiang, China; ^4^ School of Public Health, Hangzhou Medical College, Hangzhou, China; ^5^ Laboratory Medicine Center, Department of Laboratory Medicine, Zhejiang Provincial People’s Hospital, Affiliated People’s Hospital, Hangzhou Medical College, Hangzhou, Zhejiang, China; ^6^ Clinical Research Center, Affiliated Hangzhou First People’s Hospital, School of Medicine, Westlake University, Hangzhou, Zhejiang, China; ^7^ Department of Hematology, Affiliated Hangzhou First People’s Hospital, School of Medicine, Westlake University, Hangzhou, Zhejiang, China

**Keywords:** acute ischemic stroke, immunophenotypic indicators, prognostic model, survival, LASSO

## Abstract

**Background and objective:**

Acute ischemic stroke (AIS) is a leading cause of mortality, severe neurological and long-term disability world-wide. Blood-based indicators may provide valuable information on identified prognostic factors. However, currently, there is still a lack of peripheral blood indicators for the prognosis of AIS. We aimed to identify the most promising prognostic indicators and establish prognostic models for AIS.

**Methods:**

484 subjects enrolled from four centers were analyzed immunophenotypic indicators of peripheral blood by flow cytometry. Least absolute shrinkage and selection operator (LASSO) regression was applied to minimize the potential collinearity and over-fitting of variables measured from the same subject and over-fitting of variables. Univariate and multivariable Cox survival analysis of differences between and within cohorts was performed by log-rank test. The areas under the receiving operating characteristic (ROC) curves were used to evaluate the selection accuracy of immunophenotypic indicators in identifying AIS subjects with survival risk. The prognostic model was constructed using a multivariate Cox model, consisting of 402 subjects as a training cohort and 82 subjects as a testing cohort.

**Results:**

In the prospective study, 7 immunophenotypic indicators of distinct significance were screened out of 72 peripheral blood immunophenotypic indicators by LASSO. In multivariate cox regression, CTL (%) [HR: 1.18, 95% CI: 1.03–1.33], monocytes/μl [HR: 1.13, 95% CI: 1.05–1.21], non-classical monocytes/μl [HR: 1.09, 95% CI: 1.02–1.16] and CD56^high^ NK cells/μl [HR: 1.13, 95% CI: 1.05–1.21] were detected to decrease the survival probability of AIS, while Tregs/μl [HR:0.97, 95% CI: 0.95–0.99, p=0.004], B_M_/μl [HR:0.90, 95% CI: 0.85–0.95, p=0.023] and CD16^+^NK cells/μl [HR:0.93, 95% CI: 0.88–0.98, p=0.034] may have the protective effect. As for indicators’ discriminative ability, the AUC for CD56^high^NK cells/μl attained the highest of 0.912. In stratification analysis, the survival probability for AIS subjects with a higher level of Tregs/μl, B_M_/μl, CD16^+^NK cells/μl, or lower levels of CD56^high^NK cells/μl, CTL (%), non-classical monocytes/μl, Monocytes/μl were more likely to survive after AIS. The multivariate Cox model showed an area under the curve (AUC) of 0.805, 0.781 and 0.819 and 0.961, 0.924 and 0.982 in the training and testing cohort, respectively.

**Conclusion:**

Our study identified 7 immunophenotypic indicators in peripheral blood may have great clinical significance in monitoring the prognosis of AIS and provide a convenient and valuable predictive model for AIS.

## Introduction

Stroke has life-threatening characteristics that is one of the leading causes of death and long-term disability in the world (1). Nearly 800 000 patients experience stroke each year in the US, nearly 700 000 of which are acute ischemic stroke (AIS). Acute ischemic stroke (AIS) is defined by the sudden loss of blood flow to an area of the brain (2–4). Biomarkers have the potential value in predicting the prognosis of AIS and improving the diagnosis and management of AIS patients, but have not yet shown sufficient sensitivity, specificity, rapidity, and accuracy. It’s reported that Benjamin Dieplinger and his team have developed novel multi-markers model that combines NIHSS, IL-6, and NT-pro BNP for simple and accurate risk stratification. Nonetheless, their multi-marker prototype approach requires further validation in an independent cohort of patients with acute ischemic stroke (5). Based on these studies, which provides us with new insights into the value of biomarkers in the prognosis of AIS. In short, there is an unmet need to accurately predict prognosis after acute ischemic stroke to guide early decision making.

Owing to blood-based biomarkers may provide additional information for identified prognostic factors. As far as we know, the number of reports on blood biomarkers for ischemic stroke prognosis has increased, but methodological shortcomings still remains (6), there are no reliable indicators to evaluate the prognosis of AIS patients (7, 8). In addition, because of the limited prognostic models available for AIS, predicting the prognosis of ischemic stroke patients remains challenging. Therefore, there is an urgent need to find new peripheral blood biomarkers or to develop prognostic models for early-stage prediction and accurate assessment of the prognosis of AIS.

*In vitro* diagnostics (IVD) has become one of the hot fields in the medical and health industry recently. It conducts *in vitro* testing and analysis of human samples (such as blood, body fluids, tissues, etc.) to obtain diagnostic information and judge the state of the body. IVD plays an important role in disease prevention, diagnosis, treatment monitoring, prognosis observation, health evaluation and prediction of hereditary diseases. Besides, *in vitro* diagnostics plays an indispensable role in chronic disease management (9).

Professor Zhang Jing has innovated and developed a stable and rapid assay method for quantitative determination of nervous system-derived plasma extracellular vesicles (EVs) in plasma, through the innovative nano-flow cytometry detection technology, and innovated the discovery of a novel peripheral blood neurogenic EVs related marker NMDAR2A, to evaluate its diagnostic value for Alzheimer’s Disease (AD) (10). However, there is not widely accepted IVD strategy for prognostic prediction of AIS. The inflammatory response after cerebral ischemia has attracted much attention in the past few years. Stroke can induce acute immune responses that involve both local and peripheral immune compartments. Immune regulation after stroke includes the accumulation of microglia and the infiltration of macrophages, lymphocytes, dendritic cells (DCs), and neutrophil streams in the ischemic hemisphere. Numerous studies have demonstrated the critical role of the cellular and humoral immune systems in post-ischemic brain injury, and the degree of neuronal damage appears to correlate with the degree of innate immune activity (11, 12). In experimental animal models such as mice and rats, the crucial functions of invading immune cells and proinflammatory cytokines have been well investigated (13–16), while the whole immune reaction pictures in human ischemic stroke is barely unknown. Even the poststroke immune regulation was mainly focused on the local lesion, such as resident immune cells and cytokines, whereas the activation in peripheral blood circulation was limited. Given the differences of systemic blood and immune responses in between animal models and human, it poses a challenge how to transform discoveries in animal models into ‘druggable’ mechanisms of ischemic stroke. Thus, studies on systemic immune responses after ischemic stroke are scarce, and whether immune responses are beneficial or harmful remains controversial. Circulating immunoassays may be promising and valuable in predicting the prognosis of AIS, however, no such attempts have been made so far.

Analysis of immune subpopulations of lymphocytes in blood is one of the most important clinical applications of flow cytometry (FCM). FCM is sensitive, rapid, and multi-parametric in its analysis and detects immunophenotypes in blood more accurately than other methods (17). To examine the value of peripheral blood immune cells in predicting the prognostic outcome of AIS, we conducted a study with a large time span and sample size. FCM was used to obtain comprehensive information on the composition, phenotype, and function of peripheral blood immune cells. The aim of this study was to develop a prognostic model of AIS using FCM and to try to understand the logic behind this model so that it can be better used in clinical application.

## Methods

### Study design and ethical approval

This was a multicenter, prospective study that recruited a total of 484 subjects from four centers across China from January 2016 to December 2021. The training cohort and testing cohort were from Zhejiang Provincial People’s Hospital, the First Affiliated Hospital of Zhejiang Chinese Medical University, Hangzhou First People’s Hospital and the Second Affiliated Hospital of Zhejiang Chinese Medical University. A disease-stratified random sampling method was used to select 255 subjects from the AIS cohort and 147 subjects from the control cohort as the training cohort for constructing the prognostic model. A further 82 subjects were selected as a testing cohort for validating the performance of the prognostic model.

All personal privacy information was well protected and removed during the analysis and publication process. This study was conducted in accordance with the Declaration of Helsinki and registered in ClinicalTrials.Gov: ChiCTR 2000040207 and approved by ethics committee of Zhejiang Provincial People’s Hospital (No. 2020QT295) and was exempt from informed consent as shown by the IRB approval letter.

### Study cohort recruitment procedure

**Figure 1:**
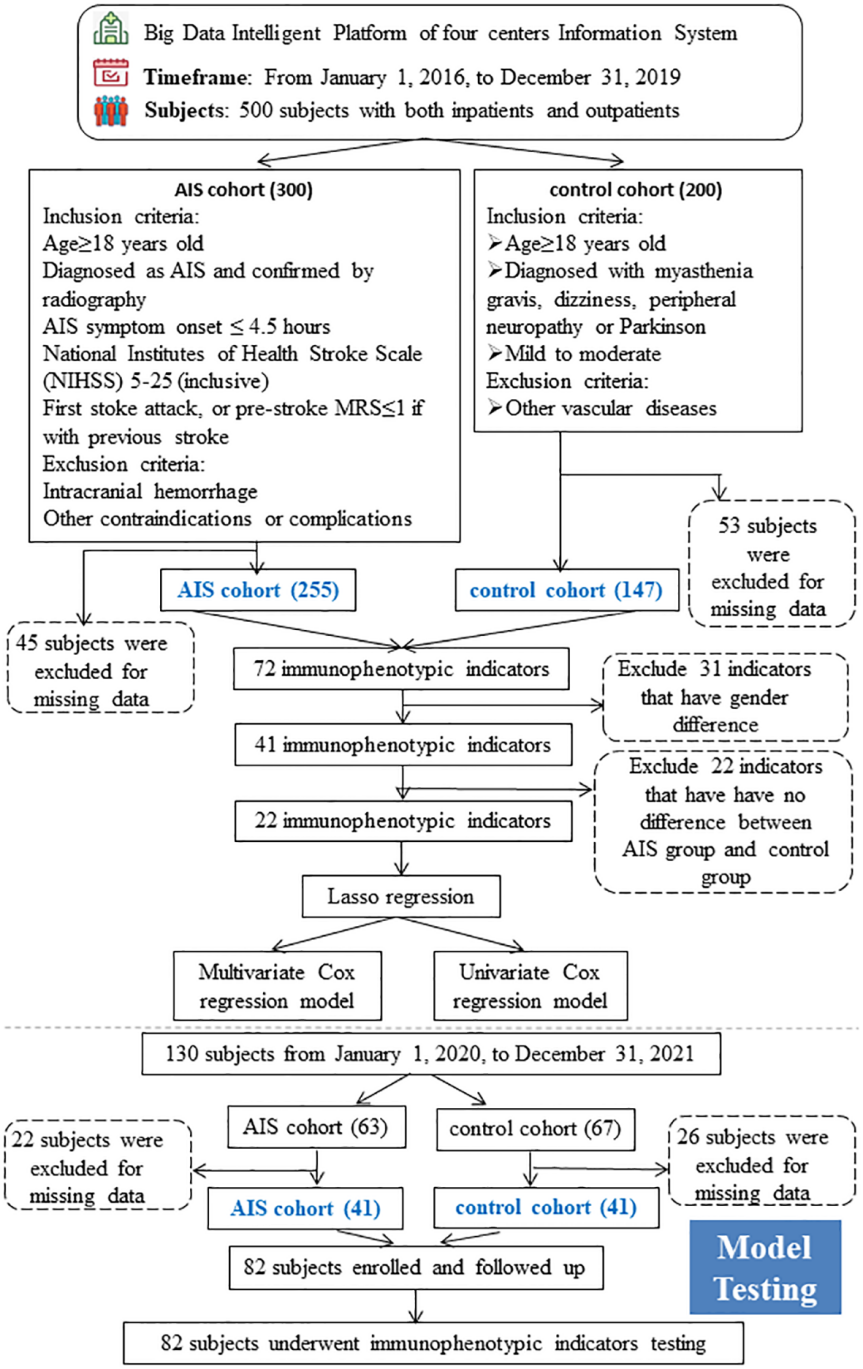
Cohort Recruitment Flowchart The whole workflow chart of this study. AIS, acute ischemic stroke.

The inclusion and exclusion criteria were detailed in the study-design workflow (**Figure 1**). Specifically, subjects with AIS were defined as those who met all the following criteria: (1) Age ≥ 18 years; (2) Diagnosis of AIS, confirmed by radiography; (3) AIS symptom onset ≤ 4.5 hours; (4) National Institutes of Health Stroke Scale (NIHSS) 5–25 inclusive; (5) First stoke episode, or those with a history of stroke with a pre-stroke MRS of ≤1. Those who with (1) intracranial hemorrhage; (2) other contraindications or complications; were excluded from the AIS cohort. Subjects with other mild neurologic but non-vascular disorders were defined as controls who met all the following criteria at the time of presentation: (1) Age is ≥ 18 years; (2) Diagnosed of myasthenia gravis, dizziness, peripheral neuropathy, or Parkinson’s; and (3) mild to moderate. Subjects with vascular diseases such as ischemic, hemorrhage, and vascular malformations were excluded as controls. All subjects were evaluated and screened by a neurologist, and 500 eligible subjects were selected, with 300 in the AIS group and 200 in the control group. 45 and 53 subjects were excluded from the AIS and control groups, respectively, due to the lack of baseline or immunophenotypic data. 255 subjects in the AIS group and 147 subjects in the control group (73 with myasthenia gravis, 40 with dizziness, 17 with peripheral neuropathy and 17 with Parkinson’s) were modeled. In addition, we additionally followed 82 subjects, including 42 AIS subjects and 42 controls, for survival status and duration after 2 years and collected their peripheral blood immunophenotypic indicators as a testing cohort for the model construction.

### Baseline characteristics

Baseline characteristics included age, sex, body mass index (BMI), systolic blood pressure (SBP), diastolic blood pressure (DBP), smoking and alcohol history, as well as medication history. Height and weight were measured with the participants standing without shoes or heavy outer garments. BMI was calculated by dividing weight in kilograms by height in meters squared. Blood pressure was detected before other parameters, including smoking, alcohol and medication history etc. were obtained by questioning during their first outpatient visit.

### Sample collection and processing

Within 24 hours of admission, the peripheral blood samples from subjects diagnosed with AIS were collected, anticoagulated with heparin and stained. Each sample was divided into five tubes and antibodies were added to each flow tube in turn according to the assay protocol, then 100 microliters of peripheral blood was taken and incubated for 15 minutes under light-free conditions, followed by 1× ammonium chloride lysate (0.15 M NH4CL, 10mM NaHCO3, 1 mM EDTANa2) mixed thoroughly for 10 minutes without exposure to light, and finally centrifuged at 500 rpm for 5 minutes, then the supernatant was removed and the sample was resuspended and mixed with 200 microliters PBS and assayed on a computer. A more detailed test procedure was described in the reference (13). The assay protocol was listed in **Supplementary Table S1**.

### Model construction

The dataset was divided into two parts: the training cohort and testing cohort. Labeled negative proportions and subgroup proportions were used as modelling features. The training cohort consisted of 255 AIS subjects and 147 control subjects, and the testing cohort consisted of 82 subjects with a 2-year follow-up.

Modeling was processed in two phases: (1) developing the model using the training queue and generating prediction estimates; (2) then validating it in the testing queue. Due to the excessive number of features, there may be some collinearity and non-significant features, so feature screening is needed to filter out the important and useful features. At the first stage, among these 72 immunophenotypic indicators, statistically significant indicators between the male and female groups were excluded to eliminate gender bias. After that, 41 immunophenotypic indicators were retained for comparison with the control group, and finally 22 immunophenotypic indicators were statistically significant with a *p* threshold of 0.05. Least absolute shrinkage and selection operator (lasso) regression, 10-fold cross-validation and penalty was used to construct the AIS prognostic model through the “glmnet” R package. Lasso regression is a widely used machine learning algorithm; compared with traditional logistic regression, it uses a penalty term, which can actively select impactful parameters from many potential multicollinearity variables in the regression, helping to reduce prediction errors.

Finally, 7 immunophenotypic indicators were selected to construct univariate and multivariate Cox regression models, and the resulting data were used to plot a nomogram to predict the probability of survival of AIS subjects. The prediction line was plotted upwards to identify the points obtained from the nomogram. The sum of these points was located on the “total points” axis; Subsequently, a line was drawn on the bottom scale to determine the probability of survival. The area under the curve (AUC) values of ROC curves were used to evaluate the performance of univariate and multivariate cox regression models. In the second stage, the cox regression model was validated and evaluated using a testing cohort of 82 subjects followed for 2 years, and the area under the curve (AUC) value of the ROC curve was used to evaluate the performance of univariate and multivariate cox regression models. At the same time, AIS subjects were divided into “high” and “low” groups based on the mean values of the 7 immunophenotypic indicators, and Kaplan-Meier curves were applied to reflect the survival differences between the two groups. The heatmap showed the seven immunophenotypic indicators used to construct the model, as well as the following clinical characteristics, including gender, age (≤60 or >60 years), the status of alcohol and tobacco use (yes, no and NA) and living status (alive, dead and NA).

### Statistical analysis

Continuous data were presented as the mean (standard deviation [SD]) or median (interquartile range [IQR]) compared by t test or Mann-Whitney test, and categorical data were reported as percentages compared by c2 test. A p-value of <0.05 was considered for statistical significance and all statistical analysis was performed with R software version 4.0.

## Results

### Demographic characteristics of cohorts

The study-design workflow was detailed in **Figure 1**. A total of 402 subjects were included in this study, including 255 with AIS, 147 with other mild neurological diseases and without any vascular diseases as controls. Baseline demographic characteristics were presented in **Table 1**. Subjects in the AIS group were predominantly males (173, 63.8%), with an older mean age (70.2 ± 13.3) and higher systolic (152.4 ± 20.7) and diastolic (81.7 ± 13.2) blood pressures than controls. In addition, the proportion of alcohol consumption (244,96.8%), use of antihypertensive drugs (157,94.0%), and lipid-lowering drugs (156,94.0%) were higher in the AIS group compared with the control group. In addition, we employed the survival plot of the group using lipid-lowering medications versus the group not using lipid-lowering medications, and the results indicated that the survival probability was higher in the group using lipid-lowering medications than in the group not using lipid-lowering medications, and we also employed a survival plot of the hypersystolic group versus the non-hypersystolic group, which showed that the hypersystolic group had a lower survival probability than the non- hypersystolic group, as was shown in **Supplementary Figure S3**. The differences between the groups of the above indicators was statistically significant (p<0.05). Body mass index (BMI) and smoking history were balanced and not statistically significant.

**Table 1:** Baseline demographic characteristics of cohorts.

Demographics	AIS group (n=255)	Control group (n=147)	*p*-value
Sex male, n (%)	173 (67.8)	69 (46.9)	<0.001
Age, years, (IQR)	71 (62,81)	58 (41.25,67)	<0.001
Body mass index, (IQR)	23.49 (21.21,25.78)	25.95 (22.25,52.50)	0.546
Systolic BP (IQR), mmHg	152 (143.25,166)	118.50 (73,138)	<0.001
Diastolic BP (IQR), mmHg	80 (73,90)	82 (71,119.25)	<0.001
Having smoking history, n (%)	80 (31.7)	20 (21.3)	0.056
Having alcohol history, n (%)	244 (96.8)	11 (11.7)	<0.001
Using antihypertension drugs, n (%)	157 (94.0)	67 (84.8)	0.018
Using lipid-lowering drugs, n (%)	156 (94.0)	67 (84.8)	0.019

a Data are n (%) or quartile (IQR). AIS, acute ischemic stroke; SBP, systolic blood pressure; DBP, diastolic blood pressure.

### Survival analysis

**Figure 2:**
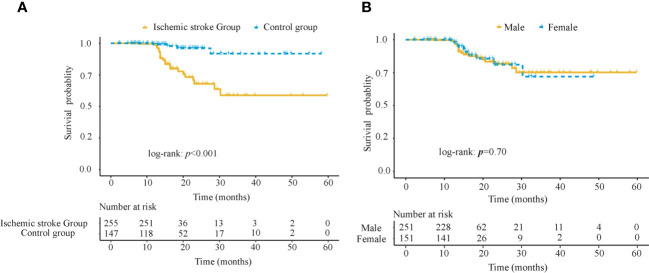
Kaplan-Meier survival analysis in 402 subjects in the training cohort **(A)** Survival probabilities between the AIS group and the control group **(B)** Survival probabilities between the male and female groups. AIS, acute ischemic stroke.

To reflect the survival differences, Kaplan-Meier curves were used to compare the survival probabilities between the AIS group and the control group, as shown in **Figure 2A**. It could be found that within 10 months, the survival probabilities of the two groups tended to be close to each other and remained around 1; then after 10 months, the survival probabilities of subjects in the AIS group was significantly lower than that of the control group, and the difference was statistically significant (log-rank: *p* < 0.001); whereas the survival probabilities of subjects in two groups tended to be equal in the next 30 months. To avoid the gender bias as indicated by the difference between the two groups, we performed a subgroup analysis to explore the survival differences between the male and the female groups, as shown in **Figure 2B**. The results showed that survival probabilities decreased in both male and female groups after 10 months and leveled off after 30 months, with no statistically significant difference between the two groups (log-rank: *p* = 0.70).

### Feature selection and model construction

**Figure 3:**
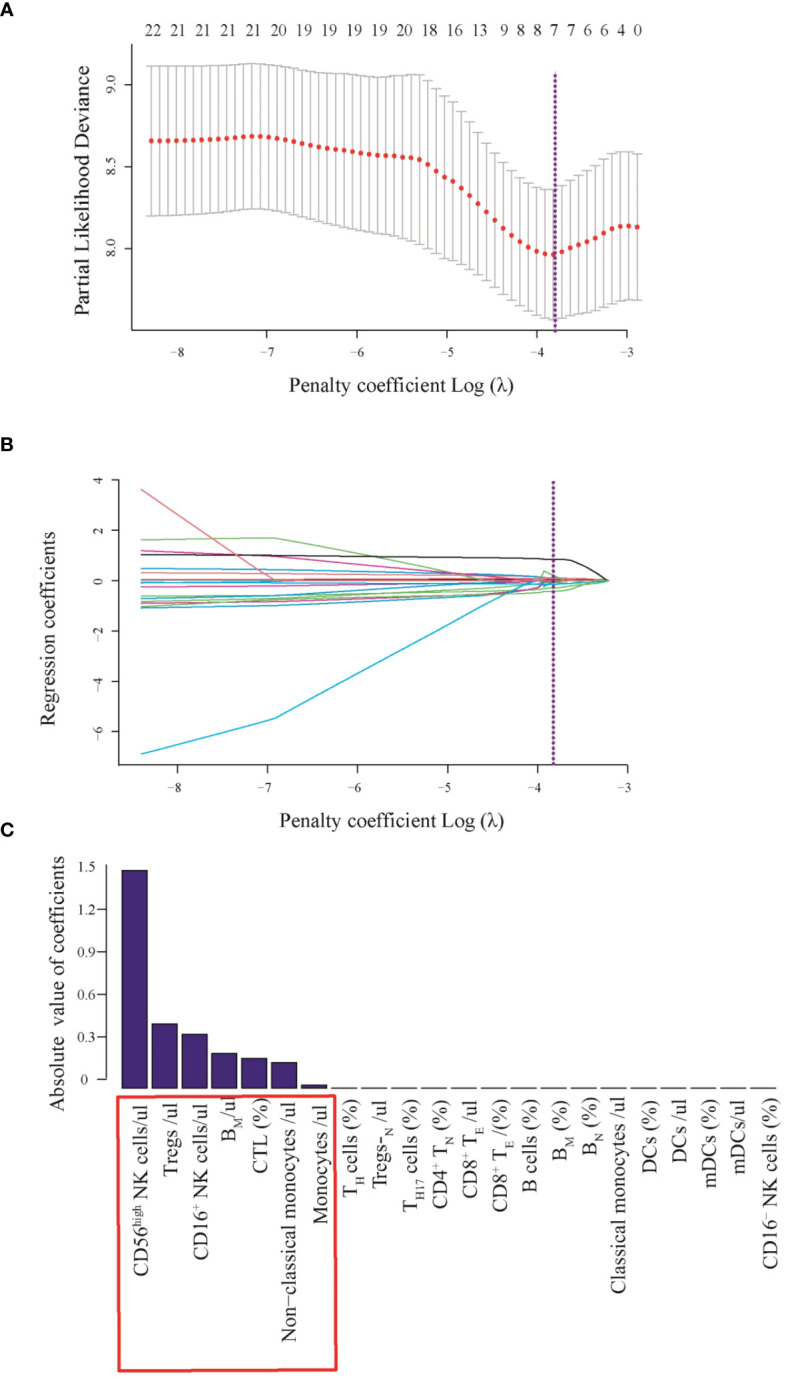
**(A)** Least absolute shrinkage and selection operator (LASSO) coefficient profiles of the fractions of 22 indicators. **(B)** Tenfold cross-validation for parameter selection in the LASSO model. **(C)** Absolute values of coefficients of 7 immunophenotypic indicators. Texture feature selection using the least absolute shrinkage and selection operator (LASSO) binary logistic regression model. **(A)** Tuning parameter (λ) selection in the LASSO model used 10-fold cross-validation via minimum criteria. The area under partial likelihood deviance curve was plotted versus log(λ). Dotted vertical lines were drawn at the optimal values by using the minimum criteria and the 1 standard error of the minimum criteria (the 1-SE criterial) A with log(λ), -3.79 was chosen (1-SE criteria) according to 10-fold cross-validation. **(B)** LASSO coefficient profiles of the 22 texture features. A coefficient profile plot was produced against the log (λ) sequence. Vertical line was drawn at the value selected using 10-fold cross-validation, where optimal λ resulted in 7 nonzero coefficients. **(C)** 7 immunophenotypic indicators with potential clinical significance which absolute value of coefficients more than zero.

**Figure 4:**
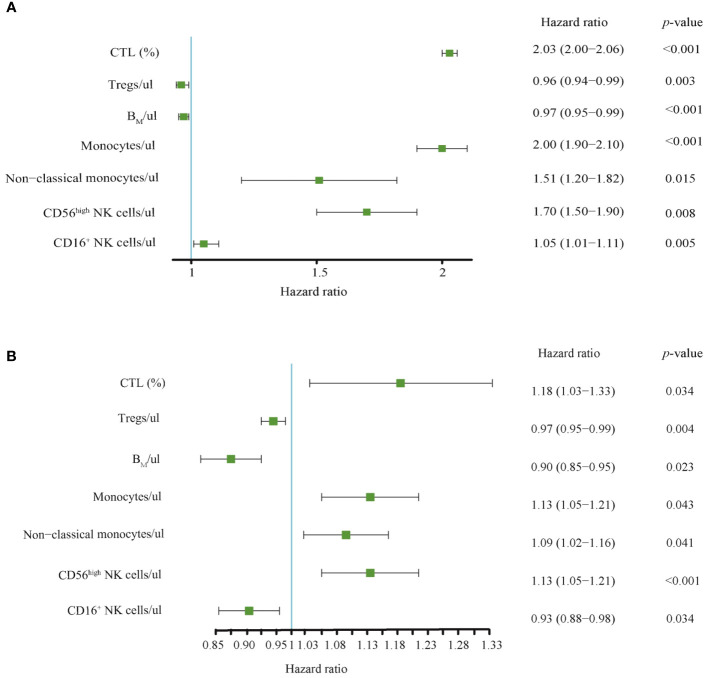
**(A)** Univariate and **(B)** multivariate Cox regression analysis for 7 selected immunophenotypic indicators. Relationship between immunophenotypic indicators and the prognosis of AIS. Forest plots was used to represent these indicators and their Hazard ratio (HR), 95% Cis, and *p*-values between AIS group and the control group. CTL, Cytotoxic T lymphocytes; B_M_, absolute number of Memory B cells.

To better understand whether systemic immune function reflects the progression and prognosis of AIS, we performed a whole-group study including 72 immunophenotypic indicators in peripheral blood samples using flow cytometry within 24 hours of AIS onset. We observed that of the 72 immunophenotypic indicators, we first excluded 31 that were statistically different between the male and female groups to eliminate gender bias. Details were shown in **Supplementary Table S2**. Then, univariate analysis of AIS versus controls was performed to screen out 22 indicators from 41 (**Supplementary Table S3**), and correlation analysis was performed on these 22 indicators, which showed that there was no strong correlation between them (**Supplementary Figure S1**). Then, the least absolute shrinkage and selection operator (lasso) regression was used to screen CD56^high^NK cells/μl, Tregs/μl, CD16^+^NK cells/μl, B_M_/μl, CTL (%), non-classical monocytes/μl and Monocytes/μl and then these indicators were used to construct prognostic model (**Figure 3**). Then, we performed univariate and multivariate Cox regression analysis to explore the immunophenotypic indicators associated with prognosis. And a forest plot was used to represent these indicators and their Hazard ratio (HR), 95% CI and *p* values between AIS and the control group, as detailed in **Figure 4**. Univariate analysis revealed that CTL (%) [HR: 2.03, 95% CI: 2.00–2.06, *p*< 0.001], Monocytes/μl [HR: 2.00, 95% CI: 1.90–2.10, p<0.001], Non-classical monocytes/μl [HR: 1.51, 95% CI: 1.20–1.82, *p*=0.015], CD56^high^NK cells/μl [HR:1.70, 95% CI:1.50–1.90, *p*=0.008] and CD16^+^NK cells/μl [HR:1.05, 95%CI:1.01–1.11, *p*=0.005] may contribute to decreasing the survival probability of AIS, while Tregs/μl [HR:0.96, 95%CI:0.94–0.99, *p*=0.003] and B_M_/μl [HR: 0.97, 95% CI: 0.95–0.99, *p*<0.001]were prognostic protective factors of AIS. On the other hand, multivariate Cox regression analysis showed that CTL (%) [HR: 1.18, 95% CI: 1.03–1.33, *p*=0.034], Monocytes/μl [HR: 1.13, 95% CI: 1.05–1.21, *p*=0.043], Non-classical monocytes/μl [HR: 1.09, 95% CI: 1.02–1.16, *p*=0.041] and CD56^high^NK cells/μl [HR: 1.13, 95% CI: 1.05–1.21, *p*<0.001] decreased the survival probability in the AIS group, and Tregs/μl [HR:0.97, 95% CI: 0.95–0.99, *p*=0.004], B_M_/μl [HR:0.90, 95% CI: 0.85–0.95, *p*=0.023] and CD16^+^NK cells/μl [HR:0.93, 95% CI: 0.88–0.98, *p*=0.034] may have a protective effect on the prognostic likelihood of survival in patients with AIS. The differences between the groups of the above indicators were statistically significant (*p*<0.05).

### Survival probability nomogram development and performance of the Cox model

**Figure 5:**
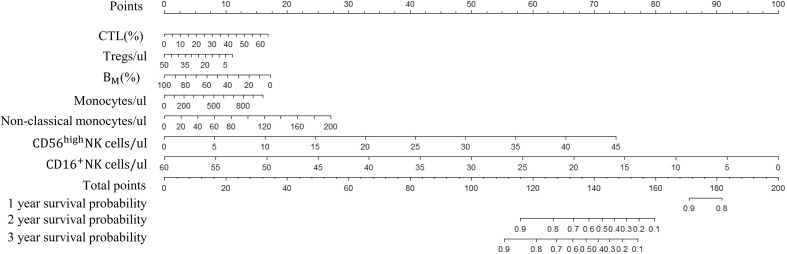
Nomogram of survival probabilities for multiple immunophenotypic indicators in subjects with AIS. AIS, acute ischemic stroke; CTL, Cytotoxic T lymphocytes; B_M_, absolute number of Memory B cells.

**Figure 6:**
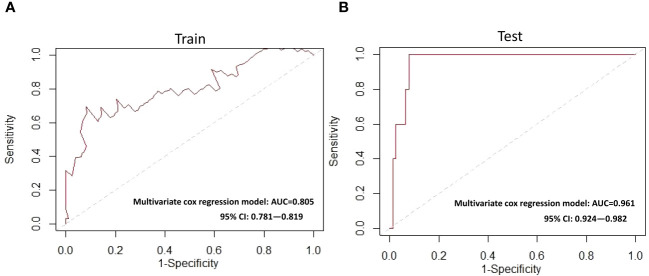
ROC curves for the multivariate Cox model **(A)** Training cohort **(B)** Testing cohort. ROC, receiver operating characteristic; AUC, area under the ROC curve.

**Figure 7:**
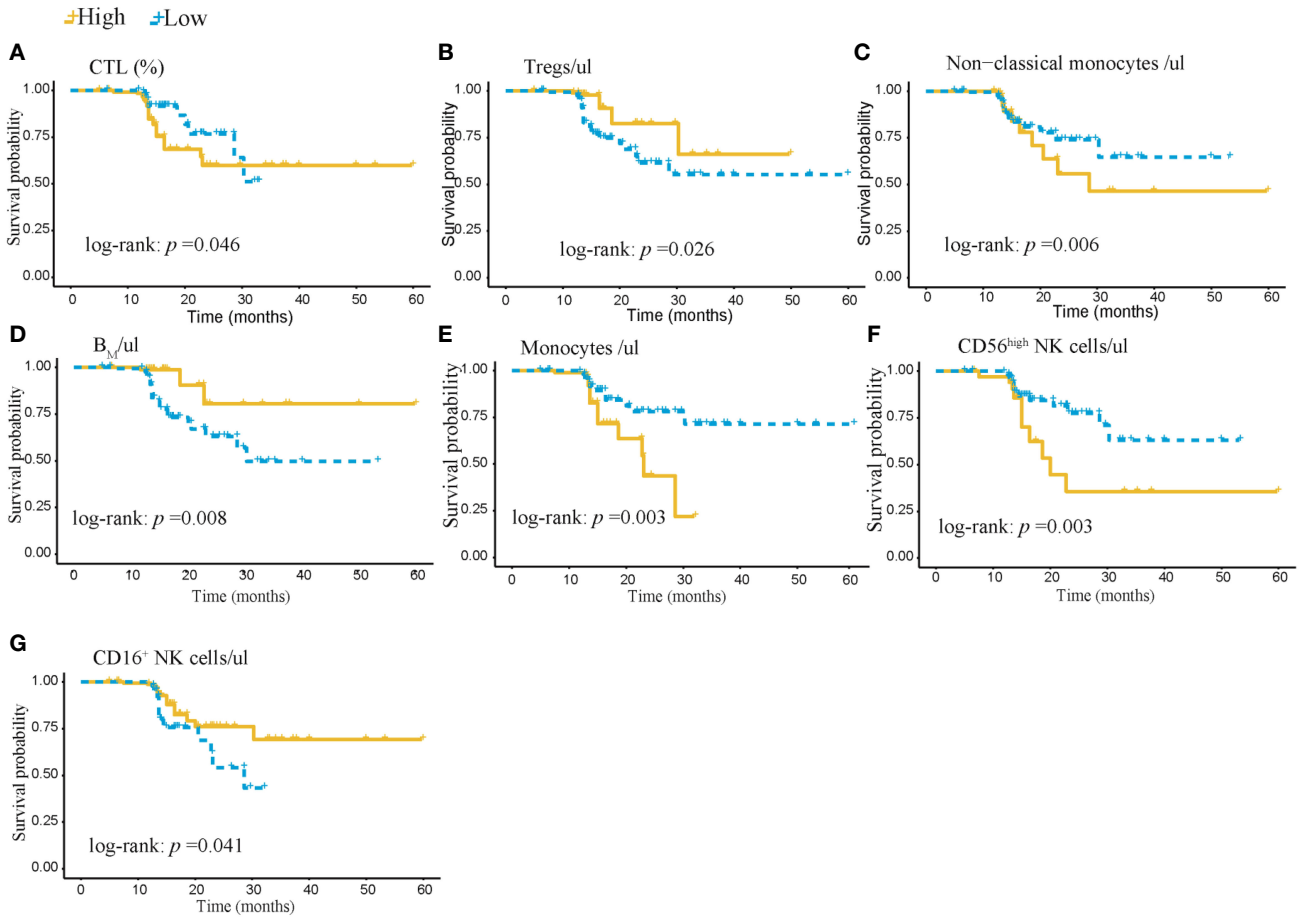
Kaplan-Meier survival analysis of 402 subjects in the AIS training cohort stratified by high and low levels of 7 immunophenotypic indicators. **(A)** CTL% and **(B)** Tregs/ul **(C)** Non-classical monocytes/ul **(D)** B_M_/ul **(E)** monocytes/ul **(F)** CD56^high^ NK cells/μl **(G)** CD16^+^NK cells/μl. AIS, acute ischemic stroke; CTL, Cytotoxic T lymphocytes; B_M_, absolute number of Memory B cells.

**Figure 8:**
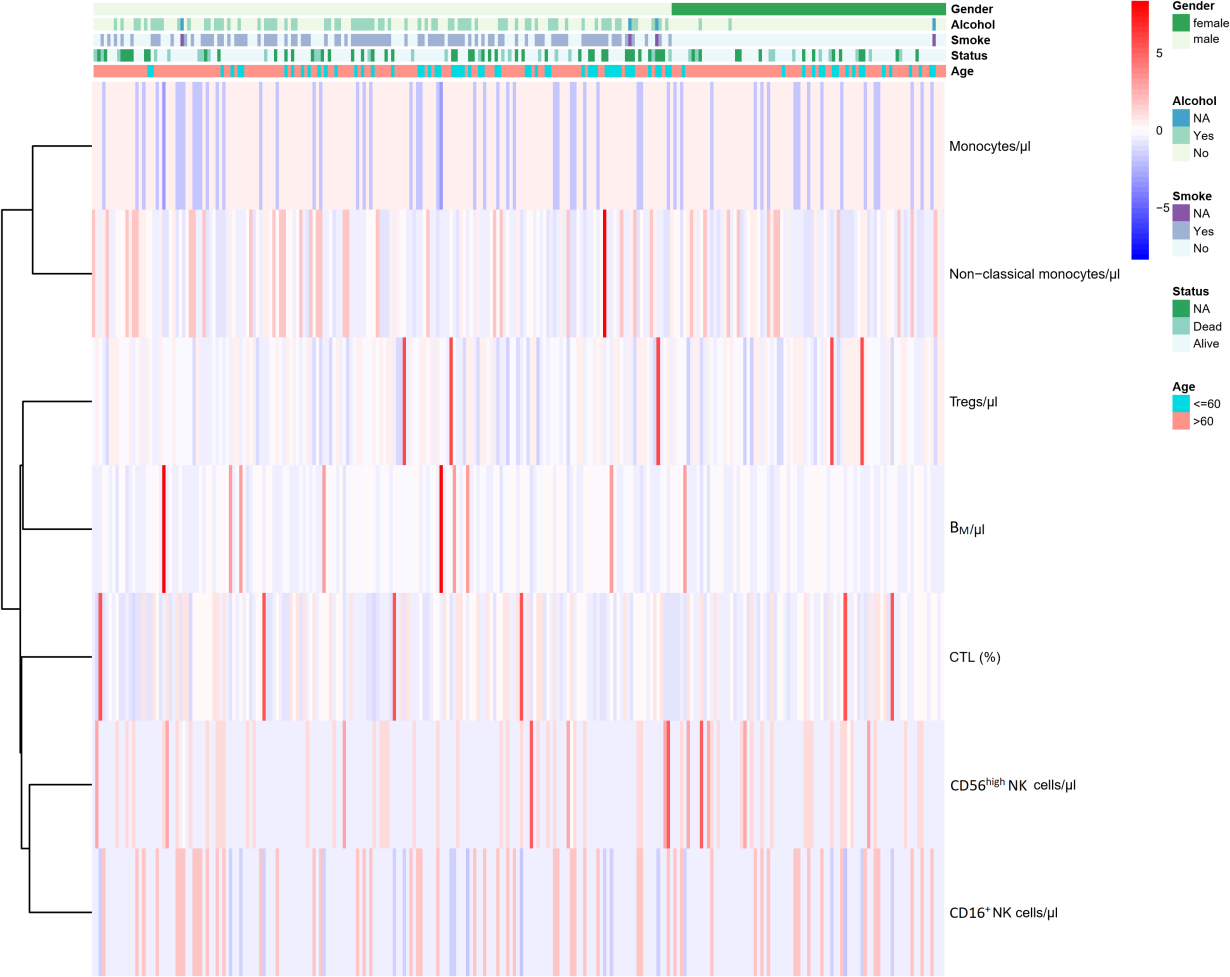
Heatmap of the relationship between the 7 immunophenotypic indicators used for model construction and the clinical features of AIS.AIS, acute ischemic stroke; CTL, Cytotoxic T lymphocytes; B_M_, absolute number of Memory B cells; NA, not available.

Based on the above 7 immunophenotypic indicators, a multivariate Cox regression model was constructed, and the 7 immunophenotypic indicators in the cox regression model were integrated into the nomogram to predict the survival rate of subjects in the next 1, 2, and 3 years, respectively. For each AIS subject, a higher total score indicated a lower probability of survival. For example, if a subject has a CTL (%) of 40, a Treg of 5μl, a B_M_ (%) of 10, a monocyte of 600μl, a non-classical monocytes of 80, a CD56^high^NK cell of 25μl and a CD16^+^NK cell of 30μl, then the corresponding points would be approximately 10, 10, 15, 10, 10, 40 and 50, respectively. The total score would be approximately 145, indicating that the estimated survival rate of this case is 48% for the next two years and 32% for the next three years. More details were shown in **Figure 5**. To assess the accuracy of predicting AIS adverse events, the AUC values of the ROC curves of the univariate cox regression model were calculated. The AUC of CD56^high^NK cells/ul was higher at 0.912 (0.884–0.954), and the AUC value of CD16^+^NK cells/μl was lower at 0.8 (0.72, 95% CI: 0.654–0.758). The accuracy of the other indicators in predicting the prognosis of AIS ranged from 0.820 to 0. 879. Detailed information on the AUC of the indicators and their 95% confidence interval was shown in **Supplementary Figure S2**. Based on the performance of these indicators in the univariate analysis, we combined the seven indicators to construct a multivariate Cox model. The results showed that the AUC of the integrated Cox model was 0.805 (0.781–0.819), which could be used to predict the prognosis of AIS. To further validate the performance of the multivariate model in predicting the survival of AIS, we followed up 82subjects for a period of 2 years and their peripheral blood immunophenotypic indicators were collected and analyzed as a testing cohort. Notably, the multivariate Cox model achieved a high AUC of 0.961 (0.924–0.982) in the testing cohort, indicating that the model was relatively stable and had a good predictive performance (**Figure 6**). Besides, we analyzed the AUC values of the four centers separately, and the AUC values of Zhejiang Provincial People’s Hospital and the First Affiliated Hospital of Zhejiang Chinese Medical University, Hangzhou First People’s Hospital and The Second Affiliated Hospital of Zhejiang Chinese Medical University were 0.712, 0.706, 0.696 and 0.764, respectively. As was shown in **Supplementary Figure S4**. To further confirm the prognostic value of these immunophenotypic indicators, we employed stratified analysis to categorize AIS subjects into high and low subgroups based on the mean value of each indicator. Notably, AIS subjects with higher levels of Tregs/μl, B_M_/μl and CD16^+^NK cells/μl had a higher survival probability than AIS subjects with lower levels. In contrast, for AIS subjects with lower levels of CD56^high^NK cells/μl, CTL (%), non-classical monocytes/μl and Monocytes/μl, survival probability may be higher than that of the high-level group (**Figure 7**). The heatmap showed the relationship between the 7 immunophenotypic indicators used to construct the model and clinical characteristics, including gender, age (≤ 60 or > 60 years), drinking and smoking status (yes, no and NA) and survival status (alive, dead and NA) (**Figure 8**).

## Discussion

Immune microenvironment and inflammatory response are involved in the whole process of AIS development (16, 18). Although the local immune microenvironment of AIS has been well studied, the relationship between AIS and systemic immunity is rarely explored. Immune cells in the central nervous system (CNS) engage in crosstalk with infiltrated peripheral immune cells, forming a complicated inflammatory network that may indirectly influence BBB integrity. Therefore, circulating, and cerebral immune cells play a profound and dual role in BBB disruption following ischemic stroke. Cerebral immune cells (CICs) and peripheral immune cells (PICs) form a subtle and complex network, and the interference may indirectly affect the integrity of blood-brain barrier (BBB) during ischemic stroke. Peripheral immune cells (including macrophages) migrate through the injured BBB to the injured site and activate host immune cells, such as microglia. Infiltrating macrophages and activated microglia release more cytokines, chemokines, and other molecules, leading to further damage or protection of the ischemic brain (11, 19). To the best of our knowledge, this is the first time to use human peripheral blood immune cell profile to establish a prognostic prediction model for AIS patients, and the sample size involved in this study is large. Previous studies mainly focused on the local inflammatory response after stroke, and few studies on the peripheral immune status. This study fills this gap.

In this study, we constructed an AIS prognostic model by considering peripheral blood immunophenotypic indicators to predict the prognosis of patients with AIS. In addition, to make our model more consistent with a natural population cohort and to avoid the failure of normal individuals to reflect the disease characteristics of AIS, we selected neurological patients from the same time, i.e., those diagnosed with non-vascular neurological disease, and age-matched cases to controls, which helped to better assess the predictive power of peripheral blood immunophenotypic indicators for AIS prognosis. We determined the relationship between overall survival (OS) and peripheral blood immunophenotypic indicators by immunophenotyping using flow cytometry. Among the seven indicators selected by Lasso, the model showed that Monocytes/μl, non-classical monocytes/μl and CD56^high^NK cells/μl were risk factors for the prognosis of AIS, while Tregs/μl, B_M_/μl and CD16^+^NK cells/μl were protective and validated by Test set. When comparing the discriminative ability, the AUC for CD56^high^NK cells/μl was the highest at 0.912; while other indicators of AIS prognosis had AUC values of 0.820 to 0. 879. Besides, based on the multivariate Cox regression model, a nomogram for predicting survival was established and the multivariate model was validated using follow-up data (AUC: 0.961). Stratification analysis revealed that the survival rates for AIS subjects with high levels of Tregs/μl, B_M_/μl or CD16^+^NK cells/μl was higher than that of the low-level group, and the survival rate of AIS patients with low levels of CD56^high^NK cells/μl, CTL (%), Non-classical monocytes/μl or Monocytes/μl was higher than that of the high-level group. The follow-up data of testing queue which contained 82 AIS patients confirmed the good accuracy and compliance of the model, and the visualized and personalized nomogram model may provide clinicians with a simple and intuitive practical prediction tool.

The exact role of monocytes in BBB disruption remains unclear, since clinical observations are controversial. Some studies suggested that their roles were harmful to BBB disruption. Previous studies indicated that monocyte counts did not change during AIS, the counts cannot predict long-term mortality (20). A recent investigation demonstrated that lower MHRs were independently associated with increased HT risk, especially sICH in AIS patients (21). Elevated levels of classical monocytes were associated with early clinical worsening and higher mortality in ischemic stroke, while non-classical monocytes were significantly decreased in stroke patients. Decreased levels of non-classical monocytes was inversely related to poor prognosis. Thus, distinct subpopulations may exert different roles in BBB integrity and stroke outcome. Ralf Stumm and his team demonstrated that Cxcr4 ablation reduces monocyte infiltration after tMCAO, which is associated with a deteriorated outcome and altered molecular responses of monocyte-derived macrophages (MDMs) and microglia (22). Intriguingly, by univariate analysis, it could be found that non-classical monocytes/ul and Monocytes/ul may be associated with reduced survival in patients with AIS. Overall, our findings are consistent with those previously reported in the literature. Circulating innate immune cells are quickly engaged at the onset of arterial occlusion, ultimately resulting in invasion of the ischemic brain by blood-borne immune cells and activation of brain-resident cells, which can be either beneficial or detrimental Monocytes enter the CSF through the choroid plexus after ischemic stroke (23). NK cells also contribute to ischemic brain injury. Yan Feng and his team indicated that miR-1224 suppresses NK cell function through Sp1 after ischemic stroke, especially in the periphery. These results suggest that blocking miR-1224 biogenesis or administering a miR-1224 antagonist might be a viable therapeutic approach for poststroke immunosuppression and infection (24). They also confirmed that miR-1224 suppressed NK cell activation and cytotoxicity specifically in the periphery rather than in the brain. Our data established that it is possible to enhance the cytotoxicity of peripheral NK cells by targeting miR-1224 while preserving the immunosuppression of brain-infiltrating NK cells to avoid aggravated intracerebral inflammation. This result is interesting and seemingly paradoxical because studies by our team and others have all suggested that NK cells can migrate into the brain parenchyma after brain ischemia (24). However, in our study, by stratification analysis, revealed that CD16^+^NK cells/μl may play a protective role and CD56^high^NK cells/μl may become a dangerous biomarker in prognosis of AIS. NK cells have been demonstrated to exacerbate brain infarction after ischemic stroke by promoting local inflammation and neuronal hyperactivity (25).

Treg cells play important immunosuppressive functions in maintaining immune homeostasis and curbing inflammatory responses in different diseases. Some studies have shown that CNS-infiltrating Treg cells, brain-resident microglia, and oligodendrocytes interact to manage white matter (WM) repair and functional recovery in the chronic stage of stroke. We have discovered that Treg cells enhance post-stroke oligodendrogenesis, at least partially, in a microglia-dependent manner. Osteopenia has been identified as a mediator between Treg cells and microglia. Boosting Treg cell numbers might be a practical and druggable approach to improve WM repair and functional recovery (26). It is apparent that the mechanisms underlying the effects of Treg cells in an ischemic brain are complicated, involving crosstalk between the CNS and peripheral immune system and interactions between Treg cells and many other types of cells. Besides, in an ischemic brain, Treg cells transmit inhibitory signals to neutrophils via PD-L1/PD-1 interactions, thereby inhibiting matrix metallopeptidase 9 (MMP-9) production and protecting the integrity of the blood-brain barrier (27). Thus, Tregs may be a promising candidate for cell-based therapies targeting post-stroke inflammatory disorders and neurovascular dysfunction. This is consistent with the results of our analysis that Tregs are protective factors in the development of AIS (28–30).

The effect of B cells on atherosclerosis has been demonstrated in many studies, and it has been established that the role of B cells is subpopulation-specific, with innate response activator and Follicular B cells promoting atherosclerosis, whereas B1 and marginal zone B cells prevent atherosclerosis (31, 32). In recent years, studies of the relationship between circulating B cell subsets and the occurrence of secondary cardiovascular events in patients with severe carotid atherosclerosis undergoing carotid endarterectomy have been reported, and their results indicated that high levels of (un)switched B memory cells are independently associated with freedom from recurrence of cardiovascular events, suggesting that patients with high numbers of (un)switched B cells may be able to prevent secondary cardiovascular symptoms in patients with severe cardiovascular disease, large numbers of unswitched and switched memory B cells are associated with a lower risk of secondary cardiovascular events (33). In addition, some researchers combined microarray profiles of stable and unstable atherosclerotic tissues from the Gene Expression Omnibus (GEO) dataset and applied the CIBERSORT method to estimate the abundance of 22 immune cell types within the tissues. Furthermore, they discussed their effects on atherosclerotic plaque instability. Atherosclerotic plaque instability contributes to ischemic stroke and myocardial infarction, and the researchers revealed the abundance and differences of immune cell types in unstable atherosclerotic carotid artery tissues. Their findings may provide some clues for future immunotherapy for atherosclerosis, as well as some new thoughts and clues for our immunotherapy research in our AIS (34, 35).

Stroke rapidly reduces the frequency of unswitched memory B cells in the circulation. It has been shown that stroke can lead to loss of B cells in experimental stroke and has been associated with post-stroke cognitive decline due to the aggregation of B cells in the ischemic brain of patients (36, 37). Furthermore, emerging research has emphasized that antibody-mediated immune damage is a key driver of post-stroke infections, in which B-cell subsets play a critical role. However, it is clear that stroke rapidly alters the dynamics of circulating memory B cells and may alter humoral responses. Whether the reduction in the frequency of unswitched memory B cells is causally related to the development of hypogammaglobulinemia after stroke remains to be further investigated (38, 39).

Taken together, these findings indeed strongly suggest a potential role for B cell subsets in the prediction and prevention of secondary cardiovascular events such as atherosclerosis. Their findings are consistent with our study that memory B cells have a protective role in AIS and are a good prognostic indicator, and high levels of memory B cells are more favorable for disease prognosis. To date, neuroinflammation is a complex event regulated by multiple factors that play a crucial role not only in the pathogenesis of the ischemic injury but also in determining its evolution. The brain insult that follows ischemic stroke results in necrosis and apoptosis; all of this drives an inflammatory reaction controlled by the discharge of ROS, chemokines, and cytokines. This process springs up in the microcirculation and involves several cytotypes, such as innate immune cells (i.e., the microglia) and adaptive immune cells (i.e., lymphocytes) causing neuronal death (40).

It has been well acknowledged that inflammation plays a key role and is involved in the whole process of acute ischemic stroke, and one of the important stages is the recruitment of leukocytes from the peripheral circulation into the ischemic tissue (41). In general, changes in peripheral blood lymphocyte subsets may reflect local inflammation in the central nervous system (42). The specific mechanism between lymphocytes and AIS may be explained by lymphocytes infiltrating ischemic tissue and mediating inflammatory responses, which increase the levels of anti-inflammatory cytokines and inhibit the production of pro-inflammatory cytokines (41). During the ischemic stroke cascade, infiltration of immune cells and release of pro-inflammatory cytokines reduce blood-brain barrier damage, brain edema, and infarct volume.

Several limitations of this study need to be acknowledged. First of all, 402 samples were recruited in this study, so larger sample trial is required to verify the relationship between peripheral blood immune indicators and the prognosis of AIS. The study participants came from 4 centers in Zhejiang Province. They were samples from a single region, so to some extent, they were not internationally representative. However, we believe that given the nature of this immunophenotypic assay model, the current approach can be implemented across different ethnic groups and regions, although the parameters of the model will be modified to some extent. Secondly, we only examined the peripheral blood immunophenotype indicators within 24hrs after AIS onset but did not monitor the fluctuations of those indicators continuously to better show the correlation with the prognosis. Third, our study confirmed the validity of peripheral blood immunophenotypic indicators in the prognostic testing of AIS, and whether the strategy can be used for other related ischemic diseases is vague. The aim of our study was to develop a validated prognostic tool for acute ischemic stroke and to confirm the performance of our model through validation.

Ultimately, an ideal biomarker for AIS should be non-invasive, user-friendly, cost-effective, and capable of detecting early signs of neurodegenerative processes before the clinical manifestations of cognitive abnormalities.

## Conclusion

In conclusion, we demonstrated the utility of a deep immune profiling approach with flow cytometry to characterize the systemic immune response comprehensively and functionally within 24 hours after AIS in peripheral blood samples. Immunophenotypic indicators of early acute stroke outcome may contribute to AIS treatment. The AIS model constructed based on the peripheral blood immunophenotypic indicators provides a new approach to predict the survival of AIS patients and provide a basis for comprehensive human studies with important implications for clinical routine. Therefore, we may provide an easily and friendly approach for the prognostic of AIS. Besides, our attempt may also lay the foundation for future systemic immunological method for AIS.

## Data availability statement

The original contributions presented in the study are included in the article/**Supplementary Material**. Further inquiries can be directed to the corresponding authors.

## Ethics statement

The studies involving humans were approved by ethics committee of Zhejiang Provincial People’s Hospital. The studies were conducted in accordance with the local legislation and institutional requirements. Written informed consent for participation in this study was provided by the participants’ legal guardians/next of kin.

## Author contributions

KL: Writing – original draft, Data curation, Writing – review & editing. WN: Methodology, Project administration, Writing – original draft. JY: Methodology, Project administration, Writing – original draft. YRC: Data curation, Methodology, Supervision, Validation, Writing – review & editing. JD: Data curation, Formal analysis, Writing – original draft. YL: Supervision, Validation, Writing – original draft. XT: Conceptualization, Data curation, Writing – review & editing. G-BC: Writing – review & editing. YW: Conceptualization, Writing – original draft, Writing – review & editing.

## Supplementary material

Supplementary Figure S1 Correlation analysis of 22 immunophenotypic indicators.

Supplementary Figure S2 The AUC values and their 95% confidence intervals for the univariate cox regression model ROC curves for the 7 immunophenotypic indicators in the testing cohort.

Supplementary Figure S3 Kaplan-Meier analyses: **(A)** Survival probabilities between the use of lipid-lowering medications and no use lipid-lowering medications groups **(B)** Survival probabilities between the hypersystolic and non-hypersystolic group.

Supplementary Figure S4 AUC curves for the four centers.

Supplementary Data Sheet 1 Flow plots of main immunophenotypes.
