# Supplementary figures and images for: Flow cytometry-based peripheral blood analysis as an easily friendly tool for prognostic monitoring of acute ischemic stroke: a multicenter study
Source: Front Immunol. 2024 May 21;15:1402724. doi: 10.3389/fimmu.2024.1402724 (PMC11148238; doi:10.3389/fimmu.2024.1402724)

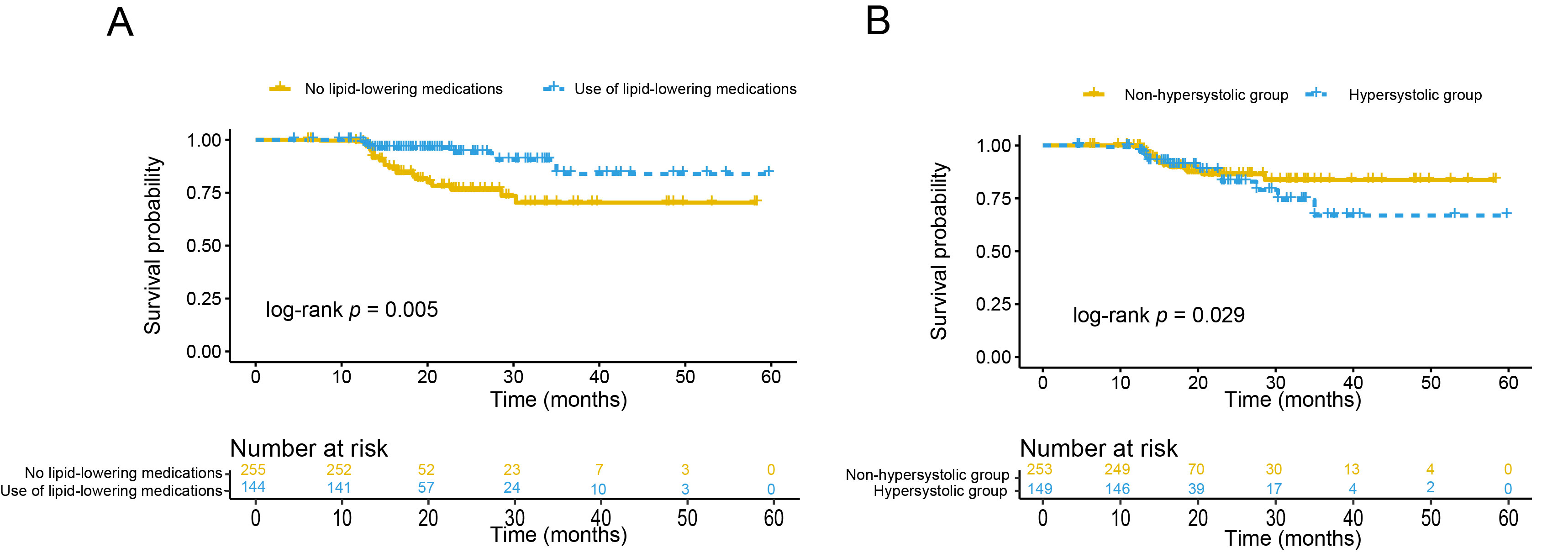

Supplement: Supplementary Figure S3 — Kaplan-Meier analyses: (A) Survival probabilities between the use of lipid-lowering medications and no use lipid-lowering medications groups (B) Survival probabilities between the hypersystolic and non-hypersystolic group. [file Image_3.jpeg]

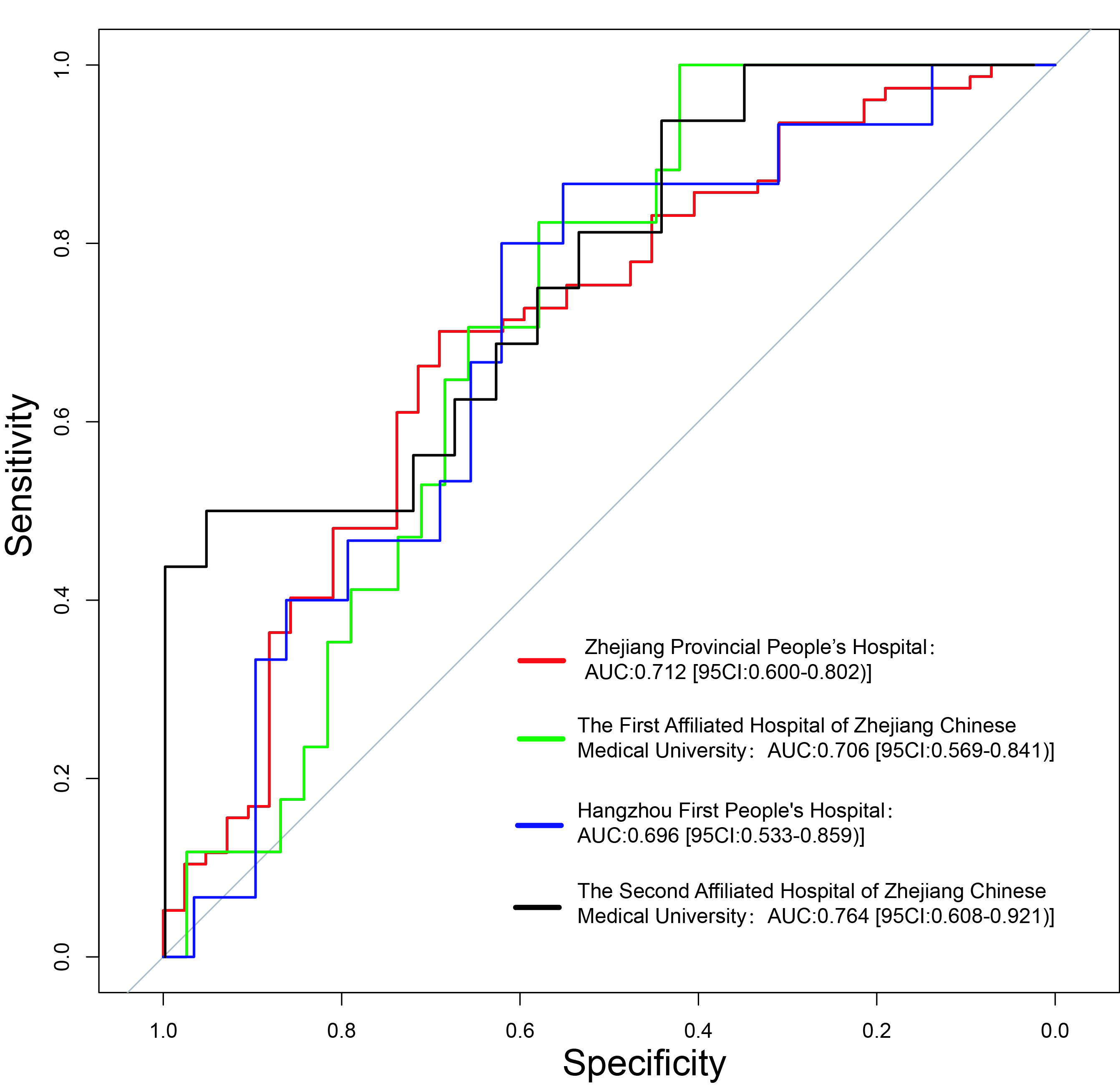

Supplement: Supplementary Figure S4 — AUC curves for the four centers. [file Image_4.jpeg]

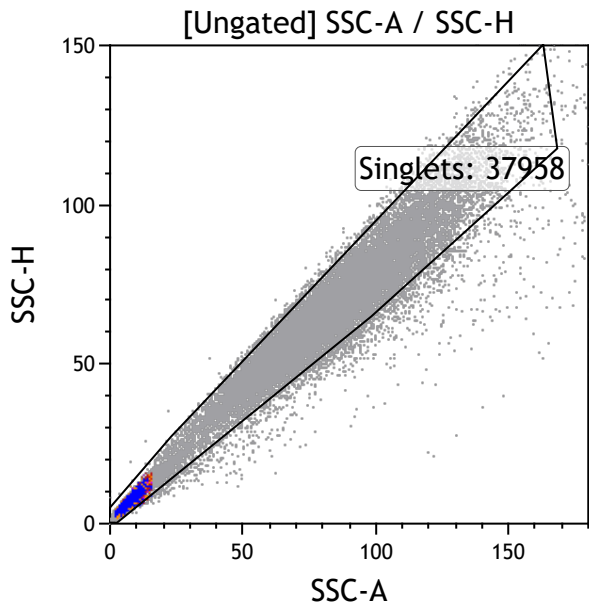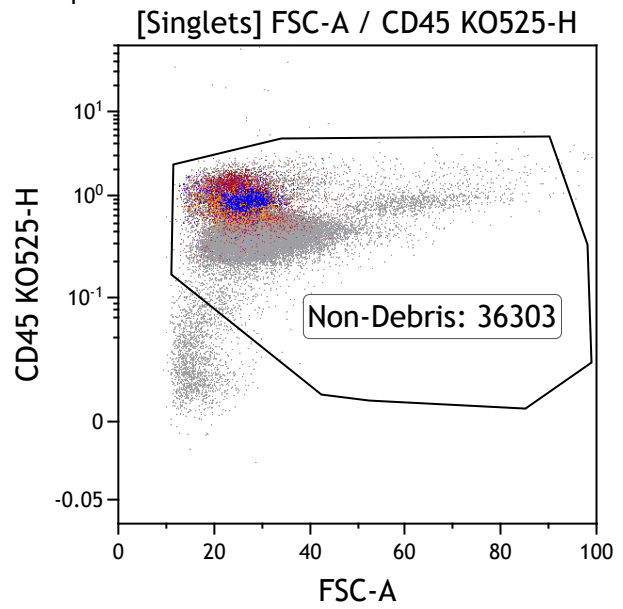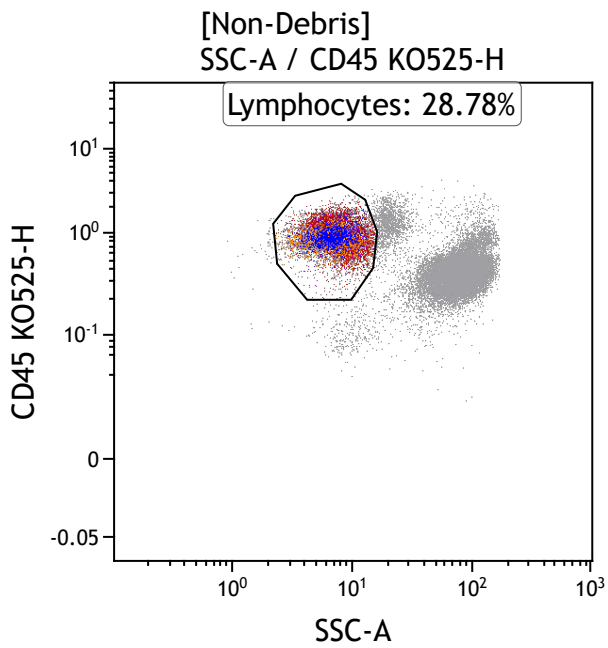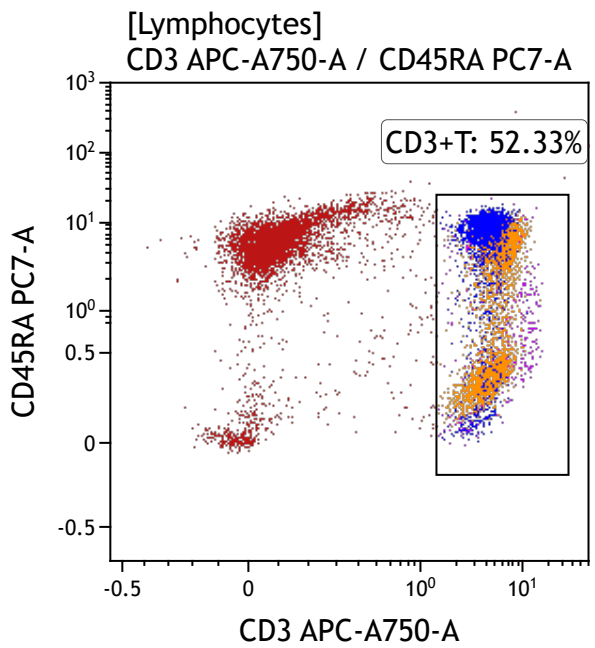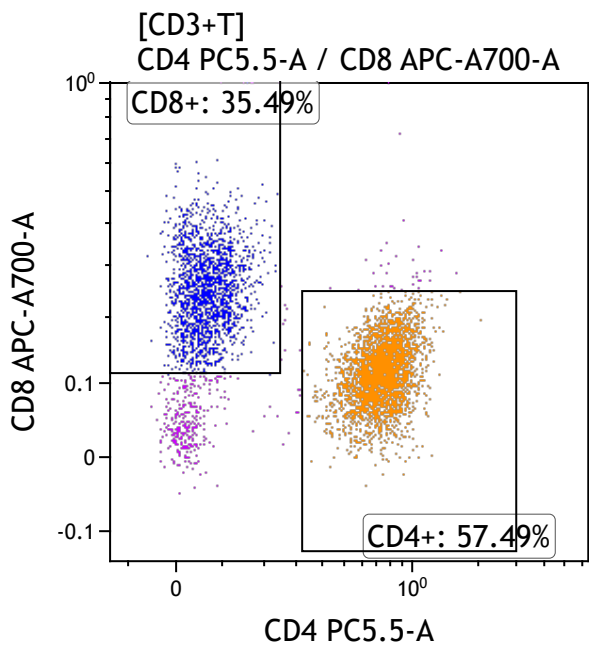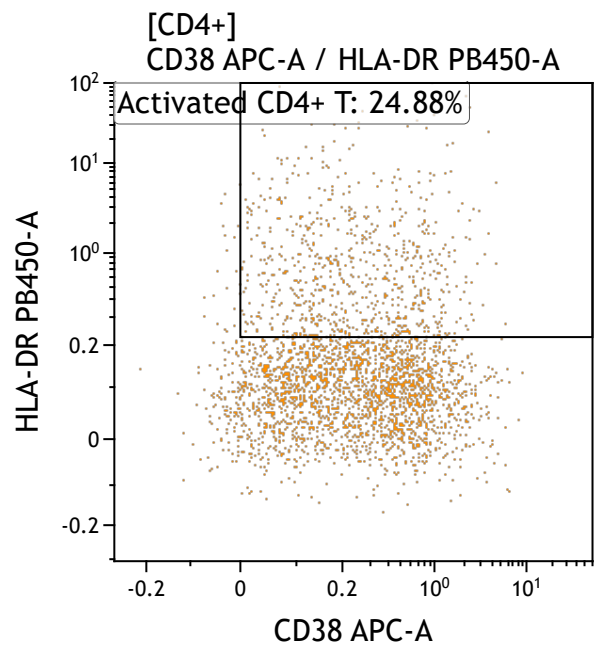

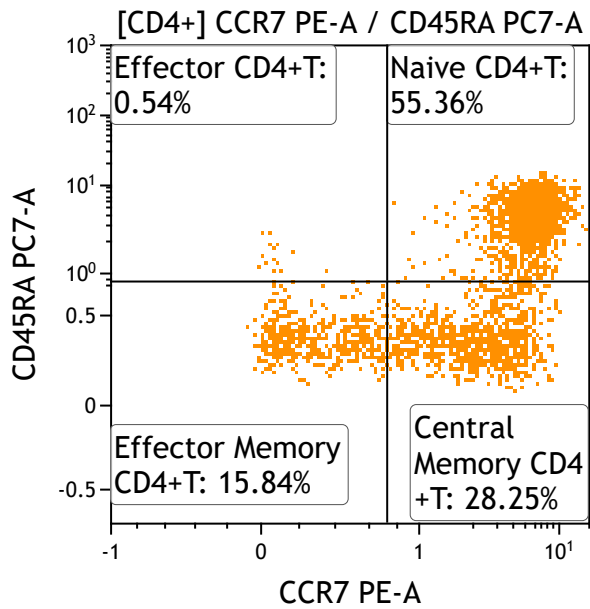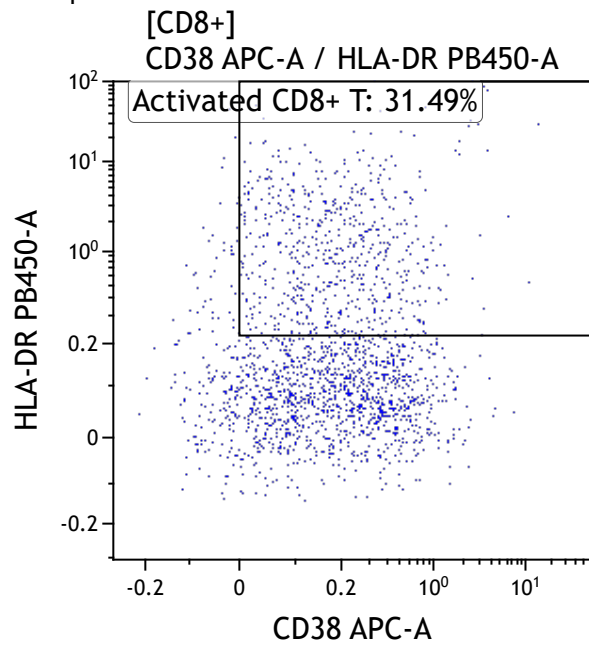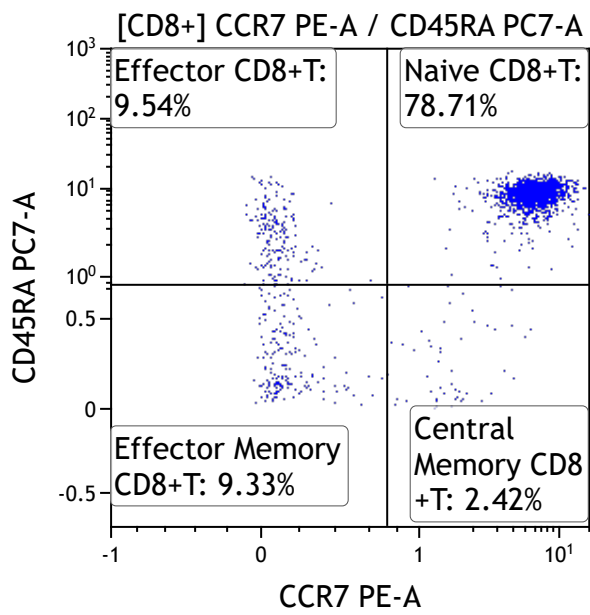

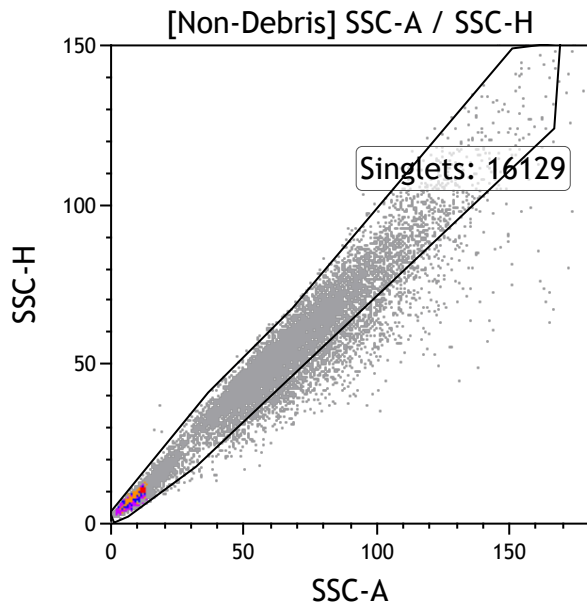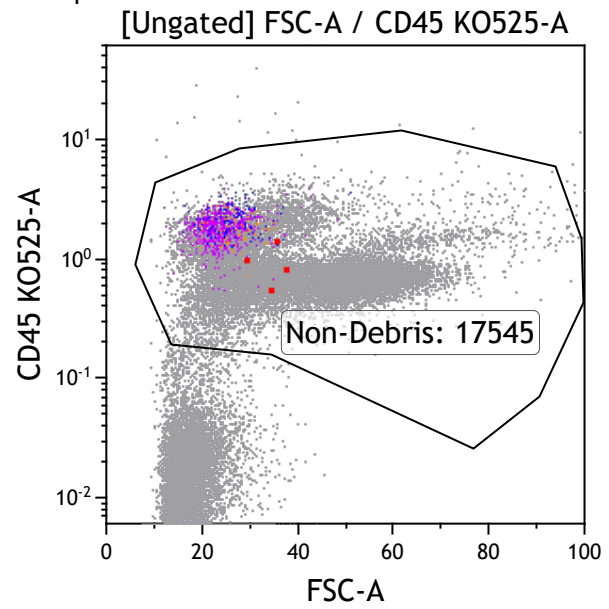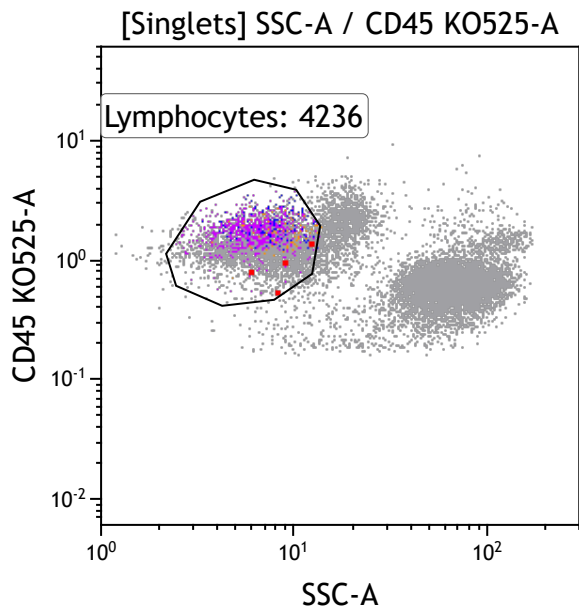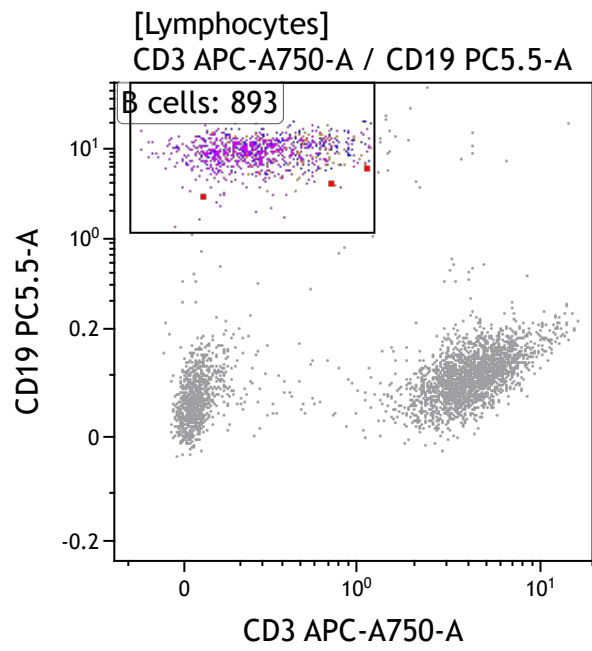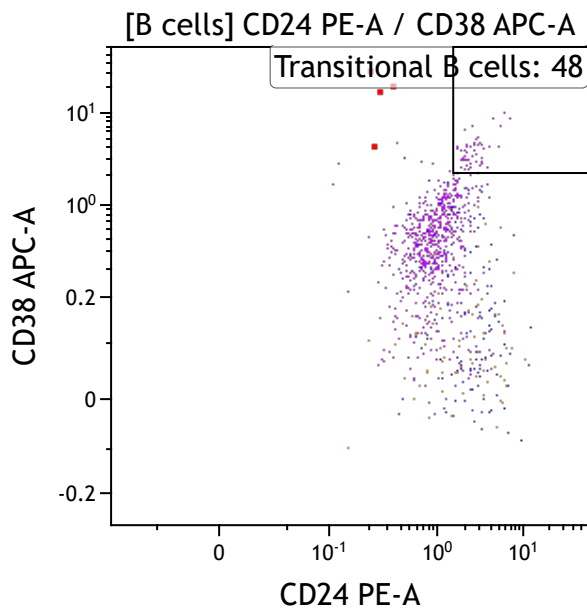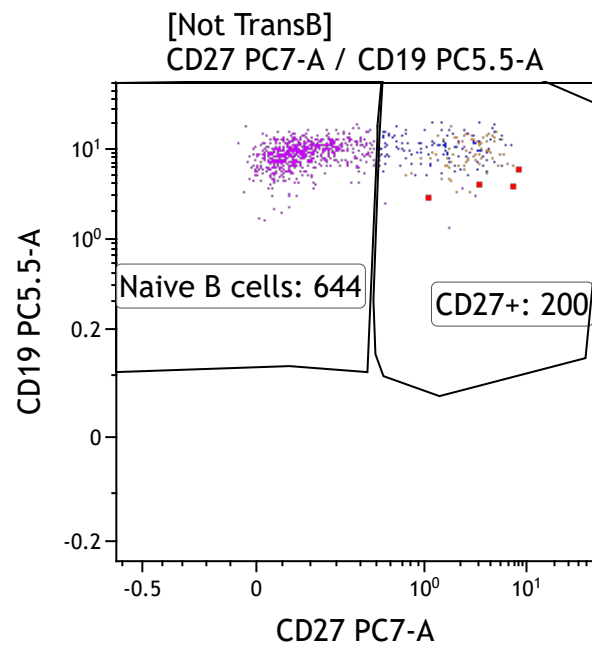

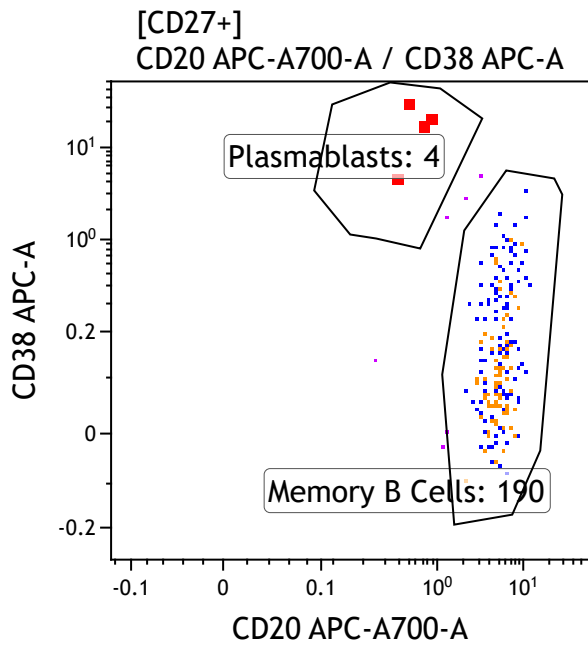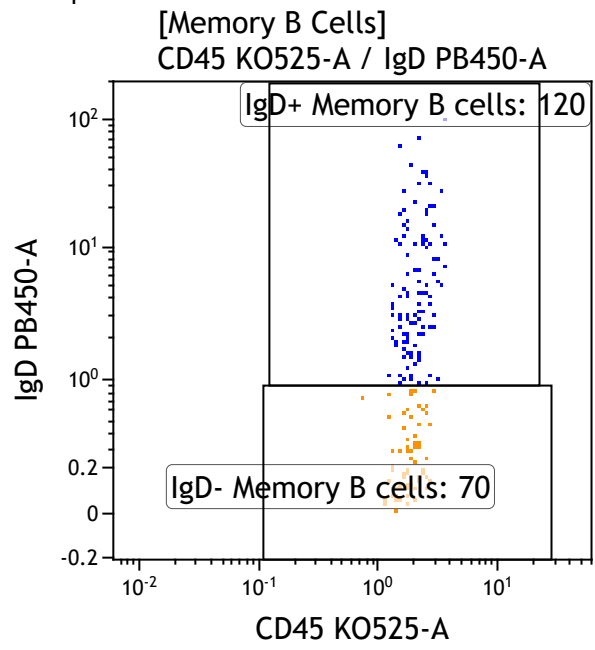

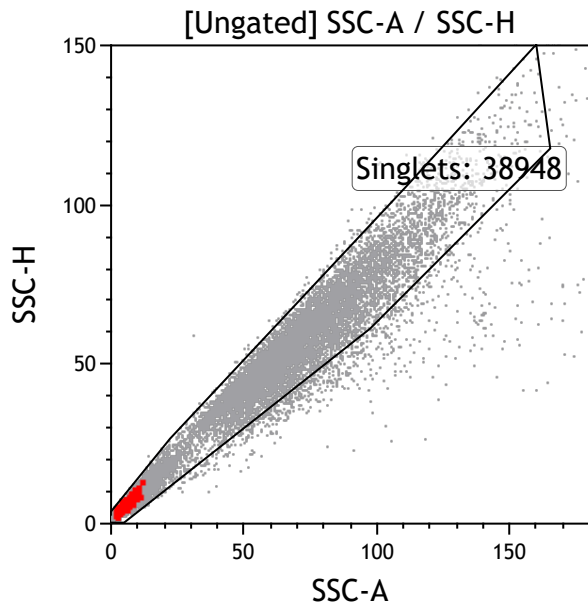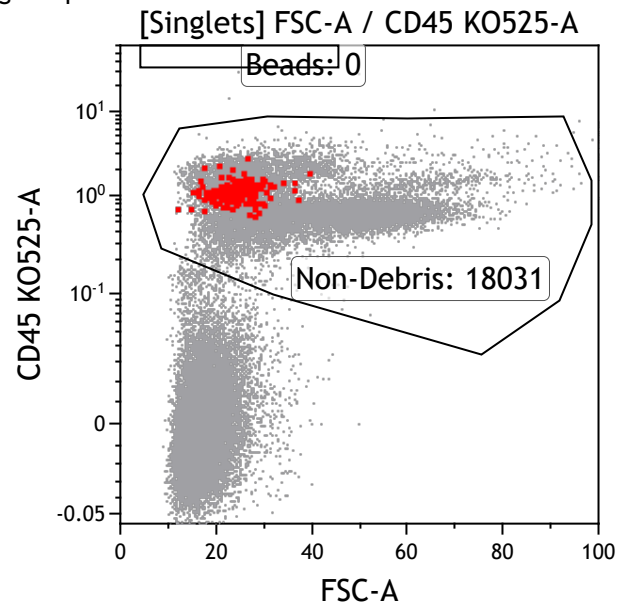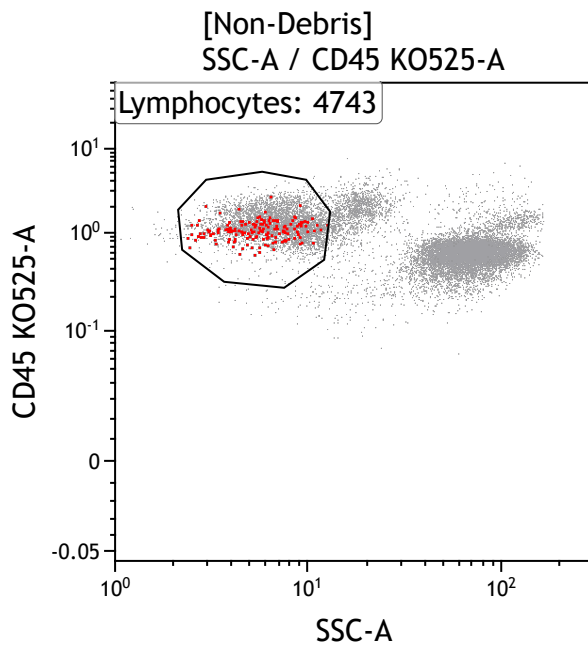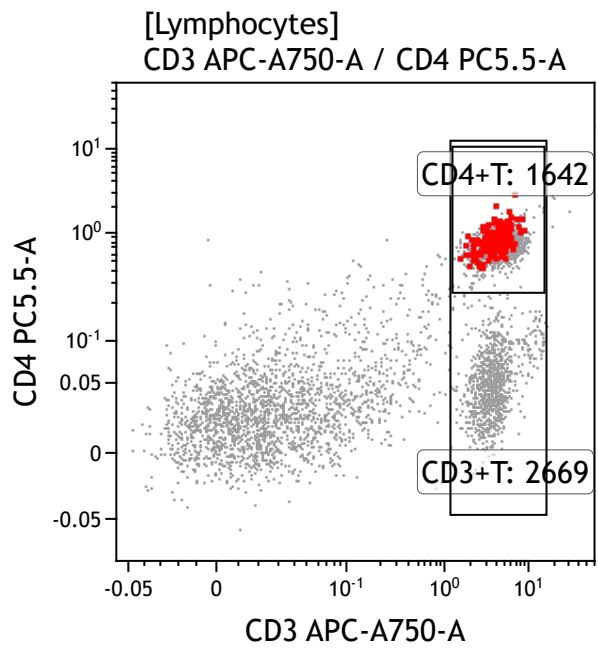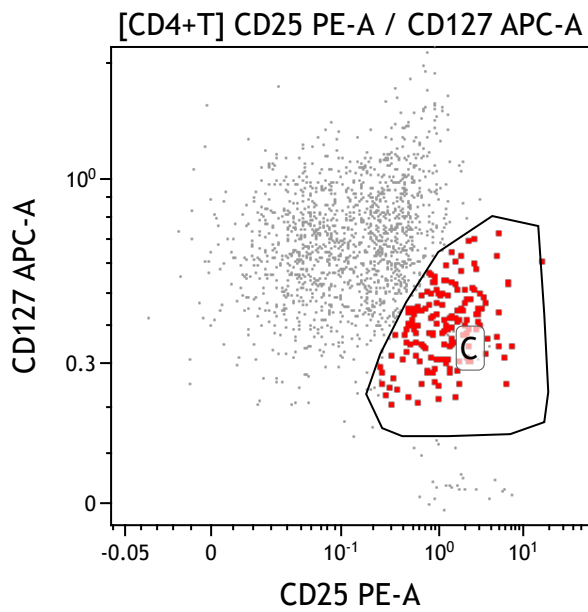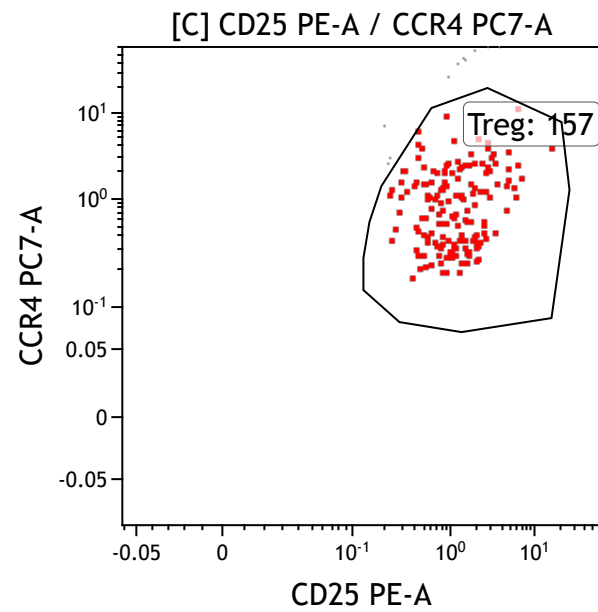

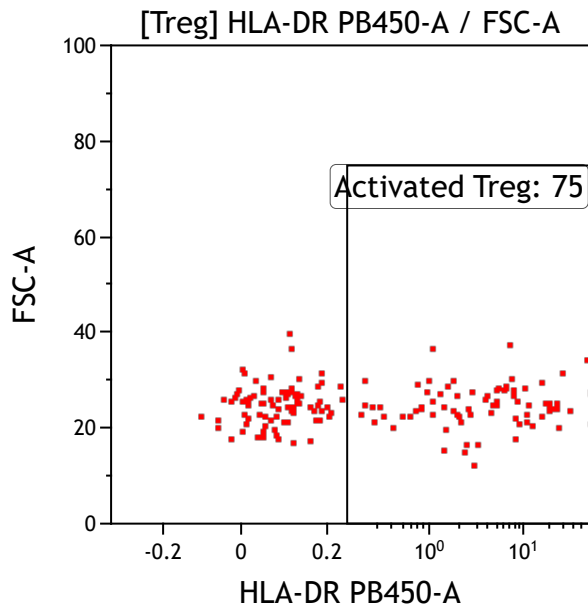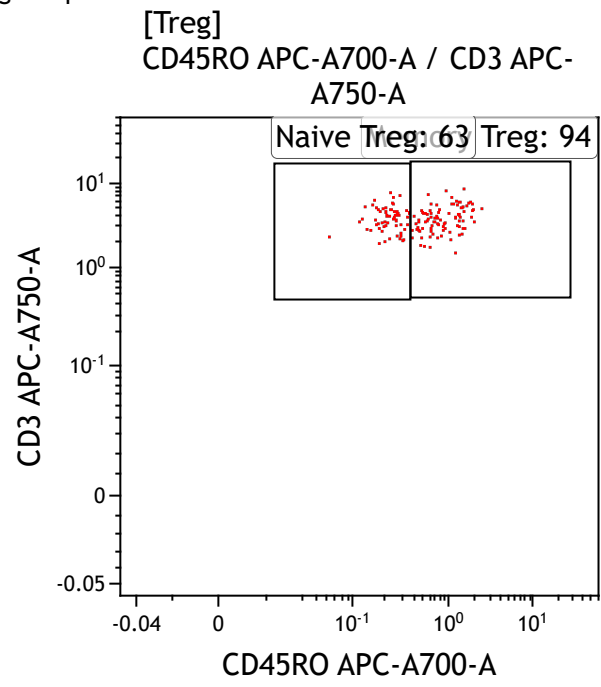

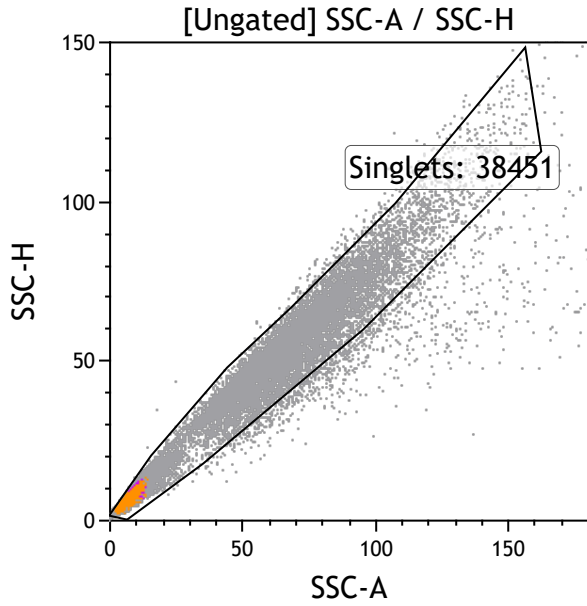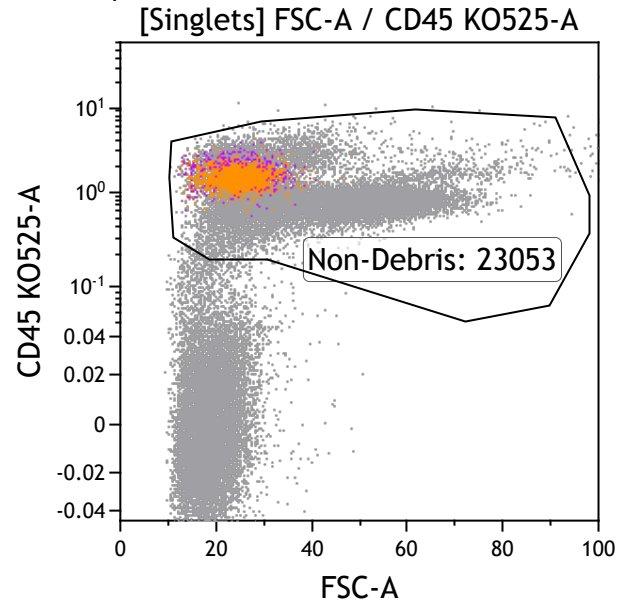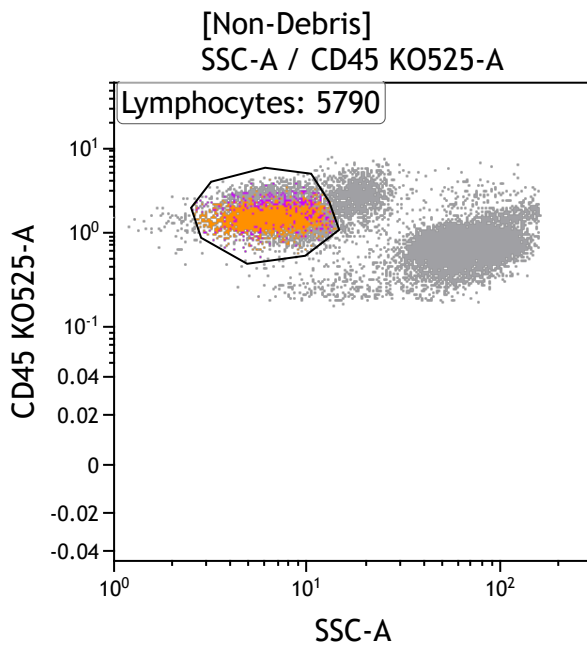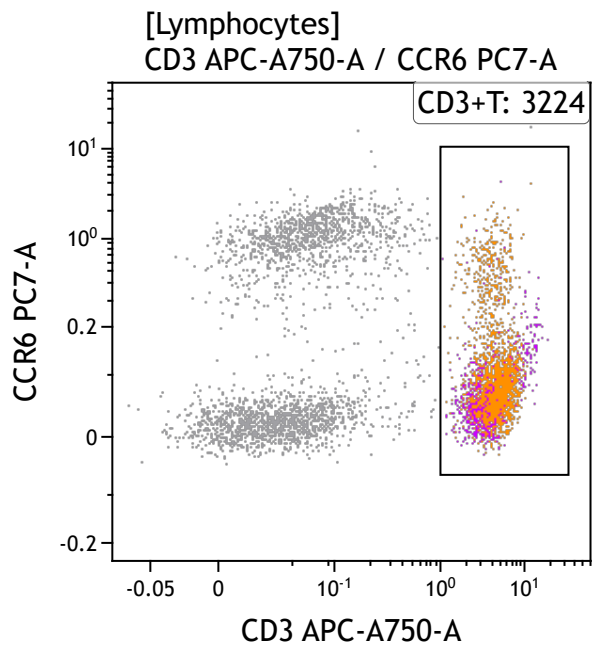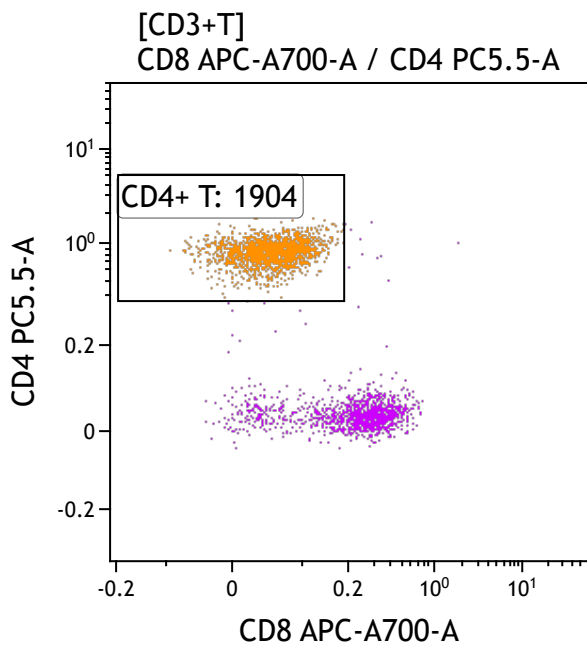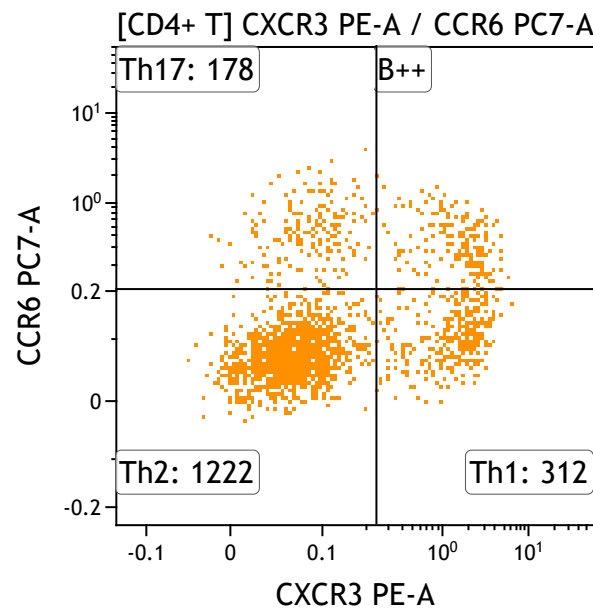

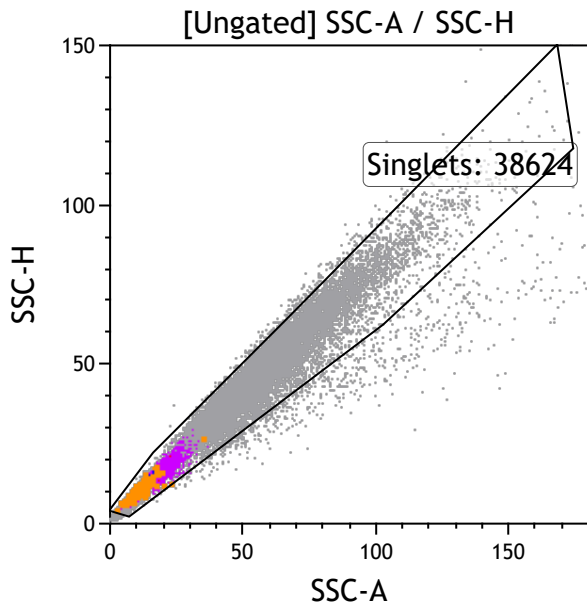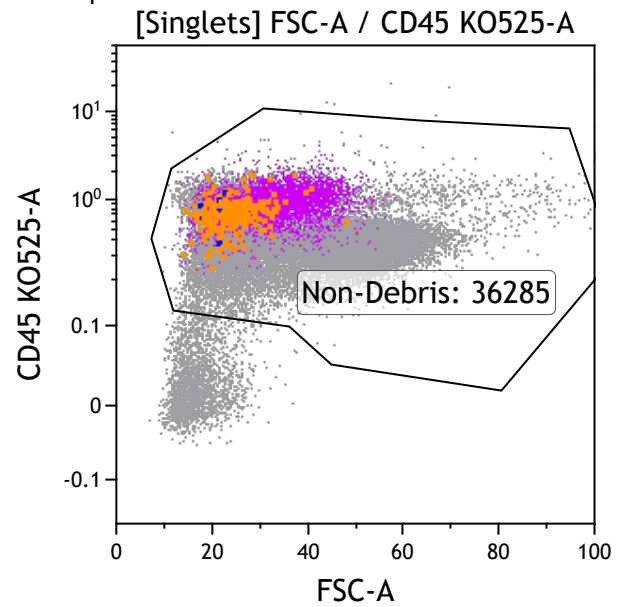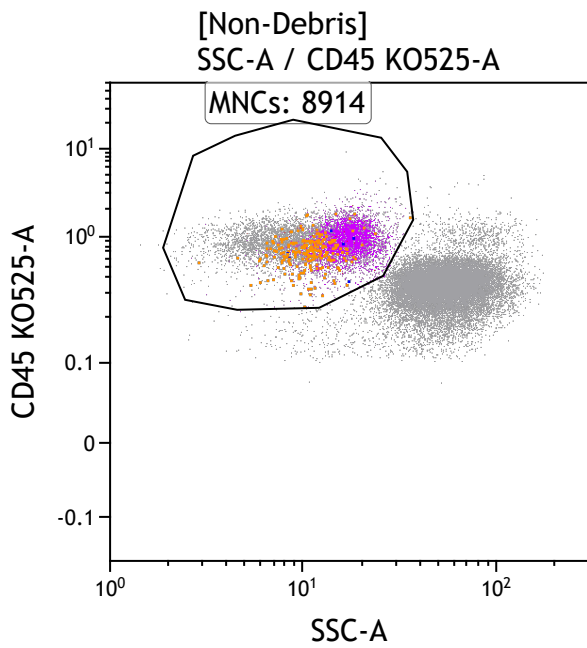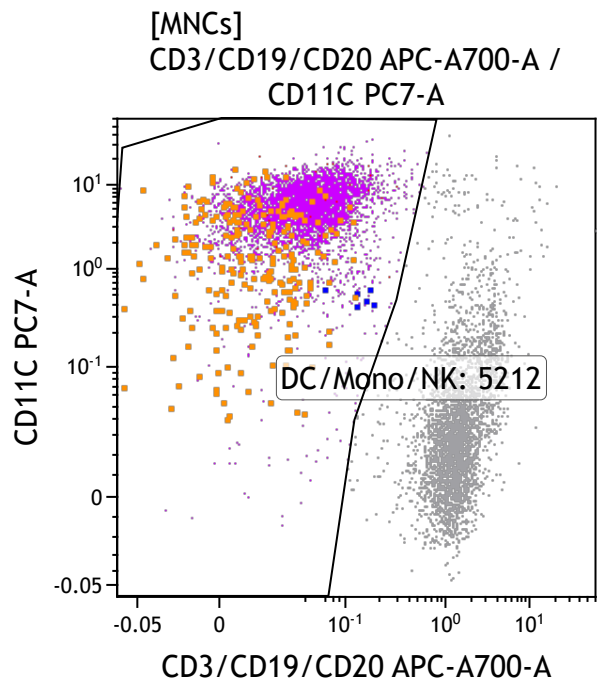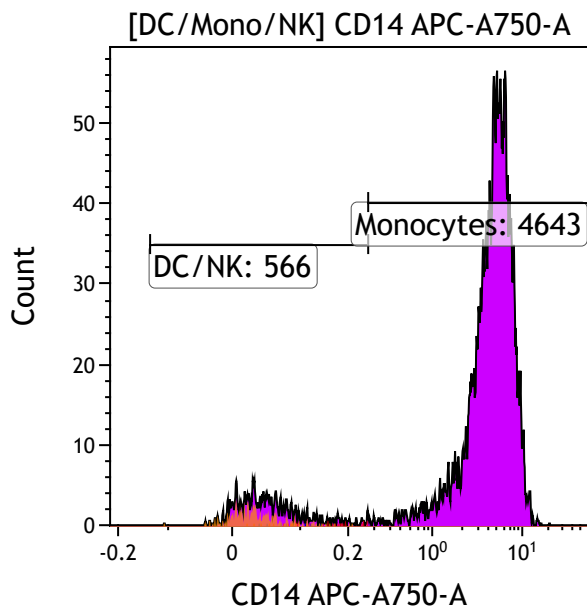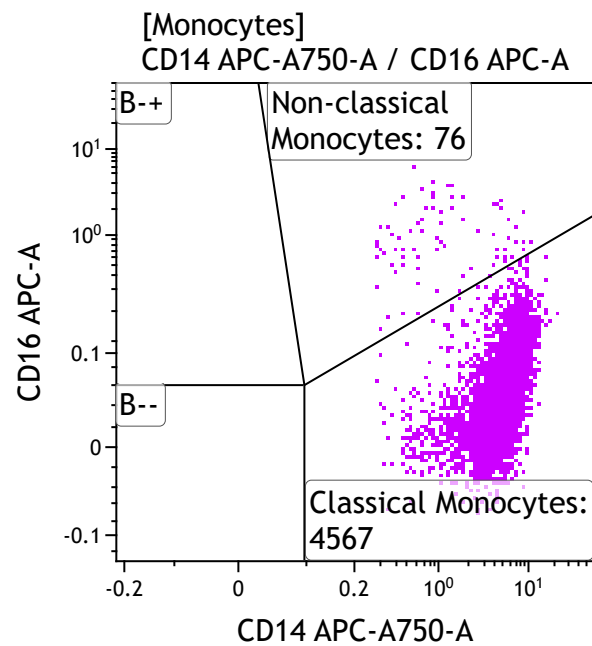

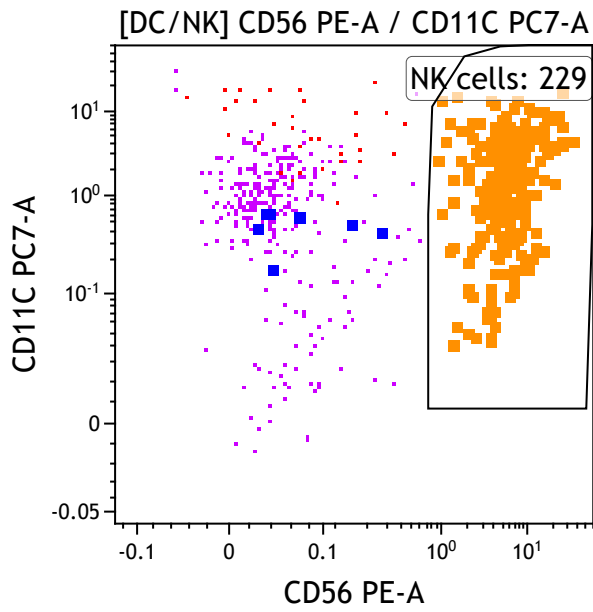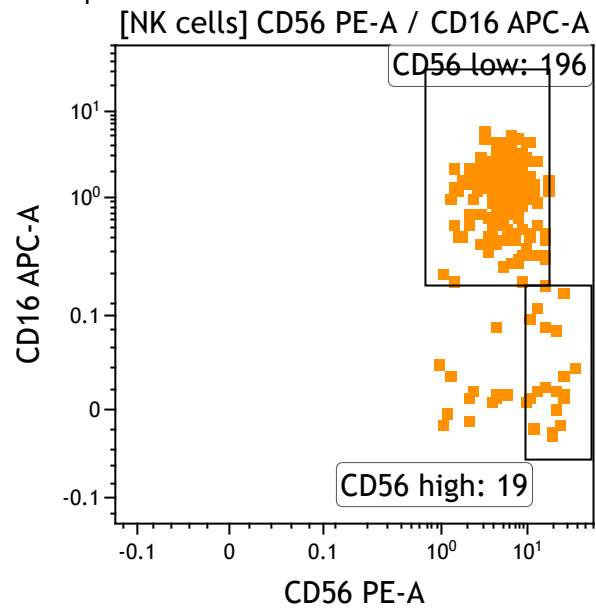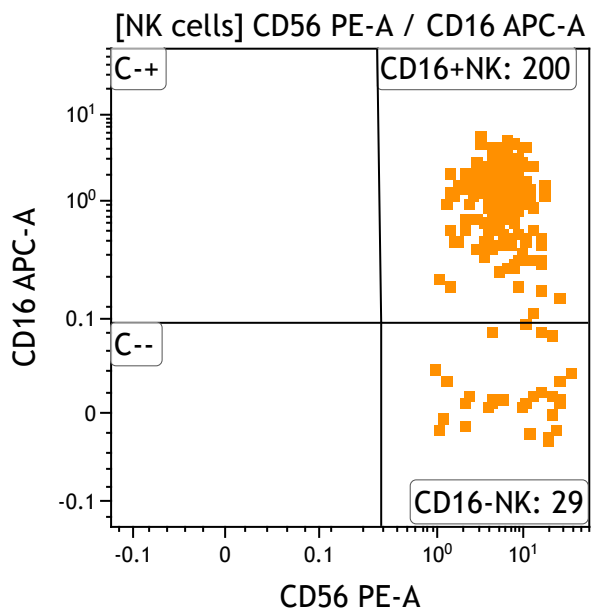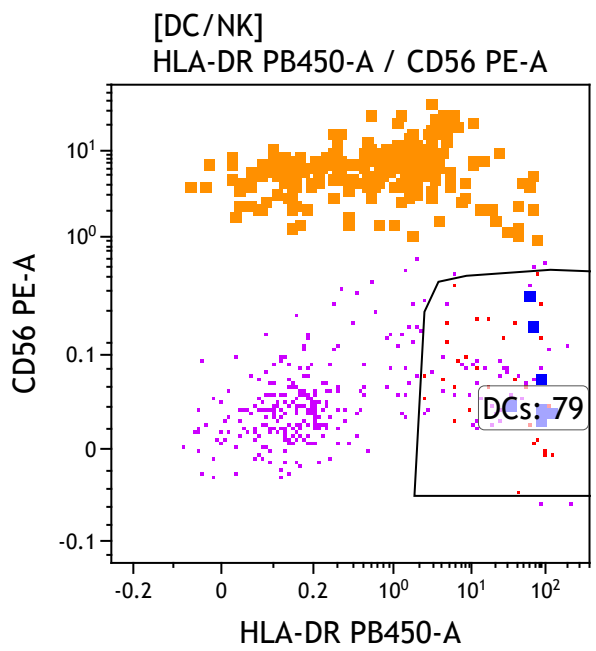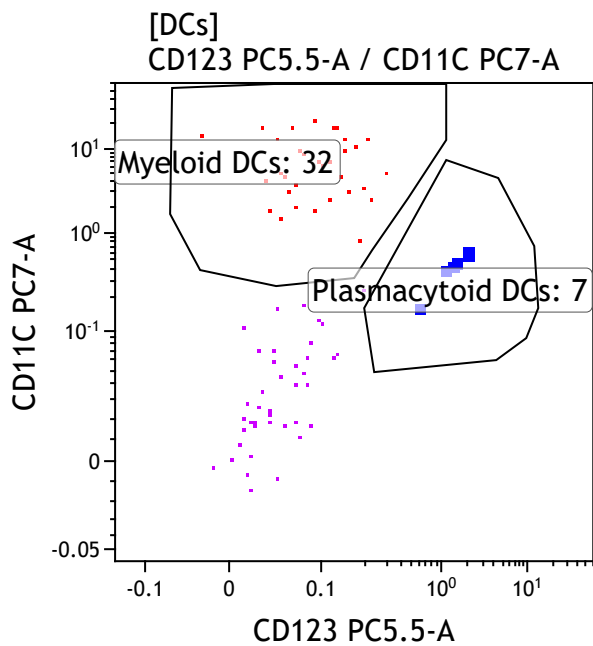

Supplement: Supplementary Data Sheet 1 — Flow plots of main immunophenotypes. [file DataSheet_1.pdf]
