# Supplementary material for: Flow cytometry-based peripheral blood analysis as an easily friendly tool for prognostic monitoring of acute ischemic stroke: a multicenter study
Source: Front Immunol. 2024 May 21;15:1402724. doi: 10.3389/fimmu.2024.1402724 (PMC11148238; doi:10.3389/fimmu.2024.1402724)
Supplement: Supplementary file 6 [file Table_1.docx]

Table S1 The assay protocol

| Fluorochrome | Marker | | | | |
| --- | --- | --- | --- | --- | --- |
|  | T cells | T_reg_ cells | T_H_1, T_H_2 and T_H_17 cells | B cells | DCs, monocytes and NK cells |
| KO | CD45 J33 | CD45 J33 | CD45 J33 | CD45 J33 | CD45 J33 |
| PE | CCR7 3D12 | CD25 2A3 | CXCR3 1C6/CXCR3 | CD24 ML5 | CD56 NCAM16.2 |
| PC5.5 | CD4 SK3 | CD4 SK3 | CD4 SK3 | CD19 J3-119 | CD123 7G3 |
| PC7 | CD45RA L48 | CCR4 1G1 | CCR6 11A9 | CD27 M-T271 | CD11c B-ly6 |
| APC | CD38 LS198-4-3 | CD127 A019D5 | CD38 LS198-4-3 | CD38 LS198-4-3 | CD16 B73.1 |
| A700 | CD8 RPA-T8 | CD45RO UCHL1 | CD8 RPA-T8 | CD20 2H7 | CD3, UCHT1  CD19, HIB19  CD20, 2H7 |
| A750 | CD3 UCHT1 | CD3 UCHT1 | CD3 UCHT1 | CD3 UCHT1 | CD14-APC-cy7 MϕP9 |
| BV421 | HLA-DR G46-6 | HLA-DR G46-6 | HLA-DR G46-6 | IgD IA6-2 | HLA-DR G46-6 |
