# Supplementary material for: Flow cytometry-based peripheral blood analysis as an easily friendly tool for prognostic monitoring of acute ischemic stroke: a multicenter study
Source: Front Immunol. 2024 May 21;15:1402724. doi: 10.3389/fimmu.2024.1402724 (PMC11148238; doi:10.3389/fimmu.2024.1402724)
Supplement: Supplementary file 7 [file Table_2.docx]

Table S2 Immunophenotypic indicators between male and female groups

| Indicators | Male | Female | *p*-value |
| --- | --- | --- | --- |
| T cells (%) | 72.77 (11.16) | 77.12 (11.16) | 0.0001 |
| T cells/ul | 884.80 (518.45) | 1075.90 (518.45) | 0.0003 |
| **Th cells (%)** | 65.32 (14.24) | 64.07 (14.24) | **0.3011** |
| Th cells/ul | 570.14 (346.52) | 667.59 (346.52) | 0.0025 |
| **CTL (%)** | 24.67 (10.42) | 26.22 (10.42) | **0.0925** |
| CTL/ul | 221.85 (176.15) | 286.73 (176.15) | 0.0003 |
| Tregs (%) | 3.25 (1.30) | 2.82 (1.30) | 0.0005 |
| **Tregs/ul** | 32.77 (23.77) | 32.04 (23.77) | **0.7563** |
| Tregs-_M_ (%) | 2.39 (1.11) | 2.04 (1.11) | 0.0007 |
| **Tregs-_M_/ul** | 23.18 (15.43) | 23.39 (15.43) | **0.9054** |
| **Tregs-_N_ (%)** | 0.85 (0.92) | 0.76 (0.92) | **0.2770** |
| **Tregs-_N_/ul** | 9.51 (15.49) | 8.49 (15.49) | **0.3806** |
| Tregs-_A_ (%) | 1.04 (0.56) | 0.88 (0.56) | 0.0034 |
| **Tregs-_A_/ul** | 10.09 (7.11) | 10.14 (7.11) | **0.9613** |
| T_H1_ cells (%) | 15.37 (5.96) | 16.96 (5.96) | 0.0031 |
| T_H1_ cells/ul | 153.28 (117.33) | 197.64 (117.33) | 0.0001 |
| **T_H2_ cells (%)** | 32.41 (12.05) | 31.10 (12.05) | **0.2123** |
| T_H2_ cells/ul | 320.51 (223.88) | 371.83 (223.88) | 0.0169 |
| **T_H17_ cells (%)** | 9.35 (4.51) | 8.57 (4.51) | **0.0750** |
| **T_H17_ cells/ul** | 85.88 (61.01) | 89.44 (61.01) | **0.5074** |
| **CD4^+^ T_N_ (%)** | 20.15 (11.20) | 19.60 (11.20) | **0.5630** |
| CD4^+^ T_N_/ul | 184.64 (162.20) | 213.77 (162.20) | 0.0492 |
| CD4^+^ T_CM_ (%) | 18.33 (11.28) | 15.03 (11.28) | 0.0006 |
| **CD4^+^ T_CM_/ul** | 150.92 (125.84) | 149.20 (125.84) | **0.2804** |
| **CD4^+^ T_E_ (%)** | 2.06 (2.87) | 2.42 (2.87) | **0.1495** |
| CD4^+^ T_E_/ul | 18.37 (28.59) | 23.20 (28.59) | 0.0483 |
| CD4^+^ T_EM_ (%) | 24.77 (11.99) | 27.02 (11.99) | 0.0326 |
| CD4^+^ T_EM_/ul | 216.20 (178.77)- | 291.42 (178.77) | 0.0001 |
| CD4^+^ T_A_ (%) | 1.55 (1.09) | 2.13 (1.09) | 0.0001 |
| CD4^+^ T_A_/ul | 13.23 (13.33) | 20.59 (13.33) | 0.0001 |
| CD8^+^ T_N_ (%) | 5.39 (5.28) | 7.43 (5.28) | 0.0001 |
| CD8+ T_N_/ul | 54.82 (87.38) | 95.28 (87.38) | 0.0001 |
| CD8^+^ T_CM_ (%) | 1.36 (1.36) | 0.96 (1.36) | 0.0004 |
| **CD8^+^ T_CM_/ul** | 11.59 (14.14) | 9.94 (14.14) | **0.1893** |
| **CD8^+^ T_E_ (%)** | 8.15 (6.66) | 8.65 (6.66) | **0.4249** |
| **CD8^+^ T_E_/ul** | 69.44 (77.28) | 78.73 (77.28) | **0.1878** |
| **CD8^+^ T_EM_ (%)** | 9.76 (7.05) | 9.18 (7.05) | **0.3114** |
| **CD8^+^ T_EM_/ul** | 86.00 (87.59) | 102.78 (87.59) | **0.0505** |
| CD8^+^ T_A_ (%) | 2.50 (2.79) | 3.49 (2.79) | 0.0010 |
| CD8^+^ T_A_/ul | 22.65 (32.53) | 38.22 (32.53) | 0.0001 |
| **B cells (%)** | 9.03(4.89) | 9.48(4.89) | **0.3061** |
| B cells/ul | 137.93(102.67) | 161.03(102.67) | 0.0310 |
| **Transitional B cells (%)** | 1.78(1.93) | 1.91(1.93) | **0.5923** |
| **Transitional B cells/ul** | 2.55(3.44) | 2.83(3.44) | **0.4139** |
| **Plasmablasts (%)** | 1.79(2.53) | 1.56(2.53) | **0.2838** |
| **Plasmablasts/ul** | 2.03(2.53) | 1.76(2.53) | **0.1664** |
| **B_M_ (%)** | 29.56(17.36) | 28.71(17.36) | **0.5602** |
| **B_M_/ul** | 38.03(38.35) | 41.27(38.35) | **0.3138** |
| **B_N_ (%)** | 68.11(17.69) | 68.70(17.69) | **0.6940** |
| B_N_/ul | 97.19(82.49) | 116.03(82.49) | 0.0342 |
| Monocytes (%) | 15.18(8.94) | 13.54(8.94) | 0.0258 |
| **Monocytes/ul** | 261.05(167.90) | 238.80(167.90) | **0.1269** |
| Non-classical monocytes (%) | 1.07(1.22) | 0.87(1.22) | 0.0319 |
| **Non-classical monocytes/ul** | 17.80(19.44) | 15.16(19.44) | **0.1084** |
| Classical monocytes (%) | 14.11(8.35) | 12.67(8.35) | 0.0379 |
| **Classical monocytes/ul** | 243.23(159.59) | 223.64(159.59) | **0.1565** |
| **DCs (%)** | 1.12(0.70) | 1.05(0.70) | **0.2900** |
| **DCs/ul** | 18.94(11.91 | 18.34(11.91) | **0.5950** |
| **mDCs (%)** | 0.74(0.54) | 0.69(0.54) | **0.3395** |
| **mDCs/ul** | 12.63(10.14) | 11.93(10.14) | **0.4513** |
| **pDCs (%)** | 0.18(0.24) | 1.17(0.24) | **0.4160** |
| **pDCs/ul** | 3.32(4.49) | 2.91(4.49) | **0.2387** |
| NK cells (%) | 10.92(6.51) | 9.16(6.51) | 0.0009 |
| **NK cells/ul** | 193.53(162.56) | 171.30(162.56) | **0.0881** |
| CD56^high^ NK cells (%) | 0.38(0.25) | 0.44(0.25) | 0.0160 |
| **CD56^high^ NK cells/ul** | 6.76(5.52) | 7.83(5.52) | **0.0504** |
| CD56^low^ NK cells (%) | 10.52(6.46) | 8.69(6.46) | 0.0005 |
| **CD56^low^ NK cells/ul** | 186.37(160.49) | 162.77(160.49) | **0.0663** |
| CD16^+^ NK cells (%) | 10.31(6.45) | 8.58(6.45) | 0.0010 |
| **CD16^+^ NK cells/ul** | 182.55(159.56) | 160.49(159.56) | **0.0846** |
| **CD16^-^ NK cells (%)** | 0.60(0.38) | 0.57(0.38) | **0.3782** |
| **CD16^-^ NK cells/ul** | 10.88(9.45) | 10.65(9.45) | **0.7742** |

Data are presented as mean ± standard error or as number and percentage, where appropriate.

Bolded text is the immunophenotypic indicators with p > 0.05, which represented the absence of sex differences.
