# Supplementary material for: Flow cytometry-based peripheral blood analysis as an easily friendly tool for prognostic monitoring of acute ischemic stroke: a multicenter study
Source: Front Immunol. 2024 May 21;15:1402724. doi: 10.3389/fimmu.2024.1402724 (PMC11148238; doi:10.3389/fimmu.2024.1402724)
Supplement: Supplementary file 8 [file Table_3.docx]

Table S3 Immunophenotypic indicators in AIS and control group

|  | Ischemic stroke | | Control group | | *p*-value |
| --- | --- | --- | --- | --- | --- |
| **Th cells (%)** | 67.21 (14.62) | | 62.67 (14.62) | | **0.00013** |
| **CTL (%)** | 2.69 (11.13) | | 26.78 (11.13) | | **0.00069** |
| **Treg cells/ul** | 29.75 (33.06) | | 34.80 (33.06) | | **0.03109** |
| Tregs-_M_/ul | 21.91 (25.18) | | 24.45 (25.18) | | 0.15479 |
| Tregs-_N_ (%) | 0.82 (0.94) | | 0.80 (0.94) | | 0.73091 |
| **Tregs-_N_/ul** | 7.77 (16.68) | | 10.19 (16.68) | | **0.03427** |
| Tregs-_A_/ul | 9.76 (15.35) | | 10.41 (15.35) | | 0.53506 |
| T_H2_ cells (%) | 31.95 (12.31) | | 31.75 (12.31) | | 0.84559 |
| **T_H17_ cells (%)** | 10.32 (4.64) | | 7.87 (4.64) | | **0.00001** |
| T_H17_ cells/ul | 89.26 (59.52) | | 85.85 (59.52) | | 0.52268 |
| **CD4^+^ T_N_ (%)** | 21.25 (10.64) | | 18.75 (10.64) | | **0.00828** |
| CD4^+^ T_CM_/ul | 156.17 (125.69) | | 136.84 (125.69) | | 0.07315 |
| CD4^+^ T_E_ (%) | 2.05 (2.80) | | 2.36 (2.80) | | 0.21597 |
| CD8^+^ T_CM_/ul | 11.65 (13.69) | | 10.19 (13.69) | | 0.24054 |
| CD8^+^ T_E_ (%) | 8.87 (7.08) | | 7.93 (7.08) | | 0.13202 |
| **CD8^+^ T_E_/ul** | 65.61 (90.68) | | 80.32 (90.68) | | **0.0357** |
| CD8^+^ T_EM_ (%) | 8.93 (7.25) | | 10.01 (7.25) | | 0.06327 |
| **CD8^+^ T_EM_/ul** | 69.22 (116.56) | | 114.20 (116.56) | | **0.00001** |
| **B cells (%)** | 9.72 (5.13) | | 8.80 (5.13) | | **0.03286** |
| Transitional B cells (%) | 1.90 (3.47) | | 1.78 (3.47) | | 0.63260 |
| Transitional B cells/ul | 2.78 (4.44) | | 2.57 (4.44) | | 0.54849 |
| Plasmablasts (%) | 1.69 (2.03) | | 1.69 (2.03) | | 0.98517 |
| Plasmablasts/ul | 1.84 (2.31) | | 1.98 (2.31) | | 0.46010 |
| **B_M_ (%)** | 27.04 (17.63) | | 31.05 (17.63) | | **0.00592** |
| **B_M_/ul** | 35.60 (40.24) | | 42.77 (40.24) | | **0.02461** |
| **B_N_ (%)** | 70.57 (17.45) | | 66.46 (17.45) | | **0.00579** |
| **Monocytes/ul** | 280.32 (164.01) | | 226.25 (164.01) | | **0.00018** |
| **Non-classical monocytes/ul** | 21.51 (13.41) | | 12.43 (13.41) | | **0.00001** |
| **Classical monocytes/ul** | 258.81 (157.45) | | 213.79 (157.45) | | **0.00101** |
| **DCs (%)** | 1.24 (0.56) | | 0.95 (0.56) | | **0.00001** |
| **DCs/ul** | 20.69 (10.83) | | 16.94 (10.83) | | **0.00089** |
| **mDCs (%)** | 0.80 (0.49) | | 0.64 (0.49) | | **0.00148** |
| **mDCs/ul** | 13.62 (9.15) | | 11.20 (9.15) | | **0.00884** |
| pDCs (%) | 0.17 (0.24) | | 0.18 (0.24) | | 0.78980 |
| pDCs/ul | 2.91 (4.64) | | 3.34 (4.64) | | 0.21196 |
| NK cells/ul | 190.58 (133.18) | | 178.01 (133.18) | | 0.33245 |
| **CD56^high^ NK cells/ul** | 6.08 (7.24) | | 8.22 (7.24) | | **0.00007** |
| CD56^low^ NK cells/ul | 184.31 (130.20) | | 168.97 (130.20) | | 0.23026 |
| CD16^+^ NK cells/ul | 181.51 (129.55) | | 165.51 (129.55) | | 0.20848 |
| **CD16^-^ NK cells (%)** | 0.53 (0.40) | | 0.64 (0.40) | | **0.00080** |
| **CD16^-^ NK cells/ul** | 8.97 (10.29) | 12.35(10.29) | | **0.00002** | |

Data are presented as mean ± standard error or as number and percentage, where appropriate.

Bolded text shows univariate analysis of AIS versus control, screening for 22 immunophenotypic indicators out of 41 indicators, *p* < 0.05.
